# Doing More with Less: Accurate and Scalable Ligand Free Energy Calculations by Focusing on the Binding Site

**DOI:** 10.1021/acs.jcim.5c02932

**Published:** 2026-02-13

**Authors:** David Alencar Araripe, Alejandro Díaz-Holguín, Antti Poso, Gerard J. P. van Westen, Johan Åqvist, Hugo Gutiérrez-de-Terán, Willem Jespers

**Affiliations:** † Department of Medicinal Chemistry, Photopharmacology and Imaging, Groningen Research Institute of Pharmacy (GRIP), Faculty of Science and Engineering, Antonius Deusinglaan 1, 9713 AV Groningen, The Netherlands; ‡ Division of Medicinal Chemistry, Leiden Academic Centre for Drug Research, 4496 Leiden University, P.O. Box 9502, 2300 RA Leiden, The Netherlands; § Department of Cell & Molecular Biology, 8097 Uppsala University, Biomedical Center, SE-75124 Uppsala, Sweden; ∥ School of Pharmacy, University of Eastern Finland, P.O. Box 1627, 70211 Kuopio, Finland; ⊥ Nanomaterials and Nanotechnology Research Center (CINN), 16379 CSIC-University of Oviedo-Principado de Asturias, and Health Research Institute of Asturias (ISPA), Av. del Hospital Universitario, s/n, ES-33011 Oviedo, Asturias, Spain; # MODSIM Pharma AI B.V., Industrieweg 9, 2254AE Voorschoten, The Netherlands

## Abstract

Predicting how chemical modifications affect drug binding is central to rational drug design. Free energy perturbation (FEP) calculations provide accurate estimates of these binding affinity changes, but existing methods often require substantial computational resources and expert knowledge. Here, we present QligFEP v2.1.0, a flexible open-source workflow based on a graphical and command-line interface for calculating relative binding free energies using spherical boundary conditions, which dramatically reduces simulation system size by confining simulations to a focused region around the binding site. QligFEP features a configurable restraint algorithm that automatically handles diverse chemical transformations, streamlined setup procedures, and enhanced analysis tools. We validated the method using industry benchmarks comprising 16 protein targets and 639 ligand transformations. Statistical analysis demonstrates that QligFEP achieves comparable accuracy to established commercial and open-source alternatives while requiring only a fraction of the computational resources. The perturbation protocol simulates ∼6250 atoms per perturbation leg and completes transformation replicates in under 2 h on standard computational clusters. Unlike full-system simulations, QligFEP’s modest computational requirements make FEP accessible for less than $1 on current AWS spot instances. The combination of accuracy, flexibility, and computational efficiency positions QligFEP as a practical solution for accelerating compound optimization in drug discovery, making rigorous binding affinity predictions accessible for large scale applications and to research groups with limited computational infrastructure.

## Graphical abstract

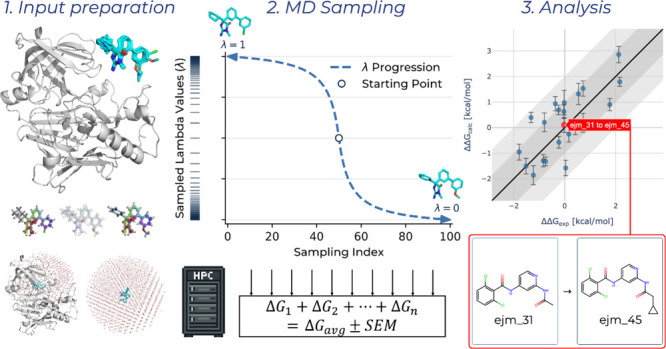

## Introduction

Relative binding free energy estimation (RBFE) methods such as free energy perturbation (FEP) have become valuable tools in drug discovery for predicting how chemical modifications affect ligand binding affinities.^1,2^ These physics-based computational methods help medicinal chemists prioritize compounds for synthesis, streamlining lead optimization. When properly parametrized, FEP methods often achieve mean unsigned errors within 1 kcal/mol,^3,4^ enabling simulation based compound prioritization in both industry and academia.^5–7^

Despite advances in computational power and force field quality,^8^ broad adoption of FEP remains limited by practical challenges: methods typically require expert knowledge for system setup, substantial computational resources, and careful quality control.^9^ Commercial platforms like Schrödinger’s FEP+ offer mature, user-friendly solutions^10^ but limit accessibility and customization. Open-source frameworks including OpenFE^11^ and pmx^12^ provide transparent alternatives with competitive accuracy.^13^ However, challenges persist around automation and computational efficiency, particularly for large perturbation networks or substantial chemical changes.

We previously introduced QligFEP,^14^ a modular workflow using spherical boundary conditions (SBC) in the Q molecular dynamics engine.^15,16^ This approach confines simulations to a focused region around the binding site, reducing system size and enabling CPU-based execution while preserving the accuracy of calculated ligand-protein interaction forces. Here we present QligFEP v2.1.0, incorporating major improvements in workflow execution, flexibility, and usability through dedicated command-line tools supplemented with graphical interface when needed. The updated platform features automated ligand mapping and network generation, streamlined input preparation, a configurable distance restraint algorithm, and enhanced analysis workflows with robust error handling. These advances reduce manual intervention, enable scripted, reproducible execution and make QligFEP suitable for high-throughput applications. We demonstrate QligFEP’s performance on comprehensive industry benchmark data sets^1,17^ and compare results to recent studies^13^ using pmx with OpenFF force fields^18^ and the commercial standard FEP+. QligFEP achieves comparable accuracy while substantially reducing computational requirements, delivering efficiency gains without compromising predictive performance.

## Methods

### Benchmarking Data and Preparation

Benchmarking data derived from the IndustryBenchmarks2024 ^19^ repository (https://github.com/OpenFreeEnergy/IndustryBenchmarks2024) comprise the JACS^1^ and Merck^17^ benchmark sets. Input structures were obtained from commit 30f6ec462f00ebc7359982cf827f45285ae2e69a, except *pfkfb3* (later added to the source repository on commit 5b7af1c4c58efdcef875a707e381bb5e94f24191). Most systems used the provided “prepared structures,” with *ptp1b* and *thrombin* prepared independently due to an unusual number of simulation crashes and poorer correlation with the experimental values when using the structures provided in the repository. Independent protein preparation was carried out starting from the same source PDB identifiers, with p*K*_a_-based protonation state assignment and local minimization.

### Structure Refinement

High-quality input structures are essential for FEP calculations. Minor adjustments resolved steric clashes and conformed structures to force field conventions. Structural manipulations, including visual inspection and energy minimization, used Schrödinger’s Maestro.^20^ Selected binding site residues underwent local minimization using integrated tools with OPLS-based force fields. For some systems (*cdk2*, *pfkfb3*), the conformation of two ligands were adjusted based on crystallographic evidence to improve alignment and correlation with experimental data (detailed in Figures S1 and S2). While not covered by our automated workflow, we recommend our users to double check rotamer orientation within aligned ligand series and to base their findings, whenever possible, on structural evidence.

### QligFEP Input Preparation

Perturbation networks were generated using scripts adapted from IndustryBenchmarks2024 (commit 30f6ec462f00ebc7359982cf827f45285ae2e69a). Kartograf^21^ mapped atoms between congeneric ligands based on Cartesian overlap, restricting transformations to same-charge pairs. Filtering functions (filter_ringbreak_changes, filter_ringsize_changes, filter_whole_rings_only) ensured sensible transformations. LOMAP scoring evaluated perturbations, generating networks with default layouts. *cdk8* required manual mapping to avoid suboptimal perturbations proposed by the automatic mapping algorithm (Figure S3).

Input file generation from SDF ligand structures and PDB protein files requires minimal user intervention. The qparams tool generates Q-compatible ligand libraries (.lib) and parameter files (.prm). The qprep_prot (CLI program creates SBC systems centered on ligand geometries, with 25 Å sphere radius encompassing binding sites. Spheres are solvated maintaining 3.0 Å minimum distance from heavy atoms. Waters clashing with ligands in specific transformations are removed during individual edge creation. Default settings include 10 replicates and automated restraint placement via the RestraintSetter algorithm.

### Force Fields and Simulation Protocol

Simulations employed Amber ff14SB^22^ for proteins, TIP3P^23^ water, and OpenFF Sage 2.2.1 ^24^ with AM1-BCC charges^25^ for ligands (openff-toolkit 0.16.4).^26^

Simulations were performed using 25 Å spherical droplets using the SCAAS surface-constrained model.^27^ Boundary waters were restrained; protein and ligand atoms outside the sphere were harmonically constrained (200 kcal/mol/Å^2^). Electrostatic interactions beyond 10 Å were approximated using the local reaction field method,^28^ except for atoms participating in alchemical transformations.

Relative binding free energies used Q’s dual topology approach. Distance restraints (0.5 kcal/mol·Å^2^) between equivalent heavy atoms maintained phase overlap, activated for distances exceeding 0.1 Å. Perturbations spanned 101 sigmoidally distributed λ windows from λ = 0.5, proceeding independently toward λ = 0 and λ = 1. Production runs sampled 10 ps per λ-window (5000 steps, 2 fs time step; total 1010 ps). Ten independent replicates per leg (protein, water) with unique random seeds ensured reproducibility—20 total replicates per edge run in parallel.

System sizes consisted of 6069 ± 92 (mean ± standard deviation) atoms for the protein leg and 6359 ± 9 for the water leg of the thermodynamic cycle (6214 ± 159 across all simulated systems, further detailed on Figure S4). Total simulation time per replicate: 1141 ps (575,000 steps including equilibration). Typical execution time consisted of 1h:36 min on CPU clusters (AMD EPYC, Intel Xeon Gold).

### Restraint Setting Algorithm

QligFEP v2.1.0 introduces the RestraintSetter algorithm (Figure 1), automating positional restraint definition for dual-topology FEP based on different levels of atom and substituent equivalency. The algorithm begins by identifying and mapping corresponding ring structures between molecules using Kartograf’s Cartesian-based atom correspondence. Following initial mapping, the algorithm hierarchically compares molecular structures using user-specified criteria:

- **Element**: Atoms are equivalent if they are the same element
- **Hybridization**: Equivalence based on hybridization state (sp2, sp3, etc.)
- **Aromaticity**: Atoms equivalent if they share aromaticity flags (RDKit definition)
- **Heavy Atoms**: All non-hydrogen atoms considered equivalent

**Figure 1:**
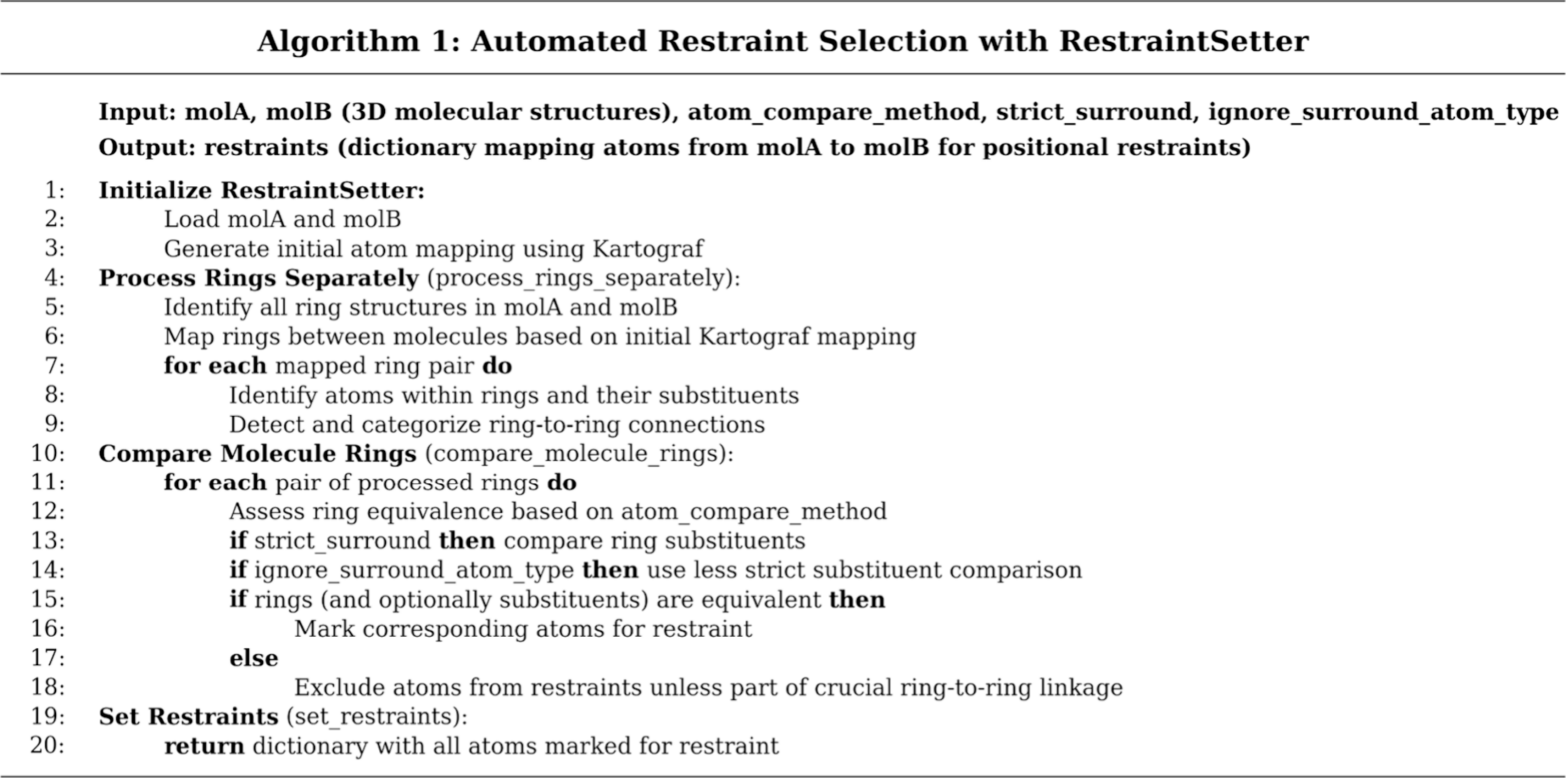
Description of the RestraintSetter algorithm. On the input, atom_compare_method is either heavyatom, hybridization, element, or aromaticity, determining the criteria for atom–atom equivalence for restraint setting. The strict_surround input is a boolean, controlling R-group equivalence checks to an element-level only. Finally, ignore_surround_atom_type corresponds to the “less strict” setting, making R-group equivalence check only for the presence of two heavy atoms.

The algorithm also considers chemical environment surrounding ring structures through three strictness levels for ring substituent comparison:

- **Permissive**: Only ring atoms compared
- **Less Strict**: Ring atoms and immediate substituents compared, element type ignored
- **Strict**: Ring atoms and immediate substituents compared, element type considered

This multitiered approach accommodates diverse molecular pairs and research needs. Restraint configurations combine atomic comparison criteria (e.g.: hybridization) with substituent strictness level (permissive = _p, less strict = _ls, strict = _s). For example, heavyatom_p treats all heavy atoms as equivalent with permissive ring comparison. Jupyter Widgets are made available to visualize restraints using py3Dmol,^29^ adapted from Kartograf^21^ and OpenFE^11^ modules.

### Analysis

The qligfep_analyze CLI computes free energies using Bennett’s acceptance ratio (BAR).^30^ For each λ window, the first 100 steps were discarded. BAR estimates for adjacent λ pairs were summed for total free energy changes. ΔΔ*G*_bind_ combined water and protein leg results via the thermodynamic cycle, with uncertainty propagated from standard errors across 10 replicates.

Performance evaluation used rank correlation (Kendall’s τ, with values ranging from −1 to 1) and mean unsigned error (MUE, average absolute deviation in kcal/mol). Metrics include 95% confidence intervals via bootstrapping (1,000 resamples) using adapted *cinnabar* methods (v0.2.3). Statistical significance employed Mann–Whitney *U* tests with Holm-Bonferroni correction^31^ via *statsannotation*.^32^ Additionally, a graphical-user interface (GUI) is available for interactively analyzing the obtained results, the perturbation networks, and the input structures used by the simulated edges (Figure S6).

Absolute binding free energies (Δ*G*) were obtained using the State Function-based Correction (SFC) algorithm for cycle closure correction.^33^ This method leverages the state function property of free energy, which dictates that the net free energy change around a closed thermodynamic cycle must be zero. Unlike traditional methods that require explicit identification of all cycles in the network, the SFC algorithm assigns an absolute binding free energy to each ligand and globally optimizes these values to minimize the discrepancy with the calculated pairwise ΔΔ*G*_bind_ values. The most connected ligand in each perturbation network was used as the reference for the SFC algorithm.

### Comparative Analysis

QligFEP performance on 16 targets was compared to results reported by Hahn et al.^13^ for pmx/GROMACS with Amber ff99SB-ILDN^34^ and Sage 2.0 ^18^ with AM1-BCC charges^25^ (PMX-Sage 2.0), and Schrödinger FEP+ with OPLS3 ^35^/OPLS3e^36^ force fields (FEP+ OPLS3e). The main comparisons discussed in this manuscript used raw ΔΔ*G*_bind_ values without postprocessing (cycle closure correction, replicate filtering) to directly assess method and force field performance.

## Results and Discussion

### Computational Efficiency of the QligFEP Protocol

A key advantage of QligFEP is its high computational efficiency achieved through SBC. By focusing simulations on the binding site region, the method substantially reduces computational requirements compared to full-system approaches while maintaining predictive accuracy. Parallel scaling analysis (2–32 CPU cores) using the *tnks2* benchmark system (Figure 2A) demonstrated good efficiency up to 16 cores. Protein leg simulations achieved 4.6× speedup (5.55 ± 0.02 h to 1.20 ± 0.01 h) and water leg simulations 4.4× speedup (5.03 ± 0.03 h to 1.15 ± 0.01 h) scaling from 2 to 16 cores, with minimal improvement beyond 16 cores. Total wall-clock time per FEP edge decreased from 105.76 h (2 cores) to 57.33 h (4 cores), 34.27 h (8 cores), and 23.46 h (16 cores), with minimal further reduction to 21.76 h at 32 cores. Eight cores provide optimal resource efficiency with 3.1-fold speedup and high parallel efficiency (77.7% protein, 76.5% water). Beyond 8 cores, diminishing returns occur, with efficiency dropping to 28.8% (protein) and 32.3% (water) at 32 cores. At current AWS spot instance pricing, a single FEP edge costs less than $1 to compute, making large-scale FEP campaigns economically feasible. We recommend using 8 cores, balancing speed and cost-efficiency.

**Figure 2:**
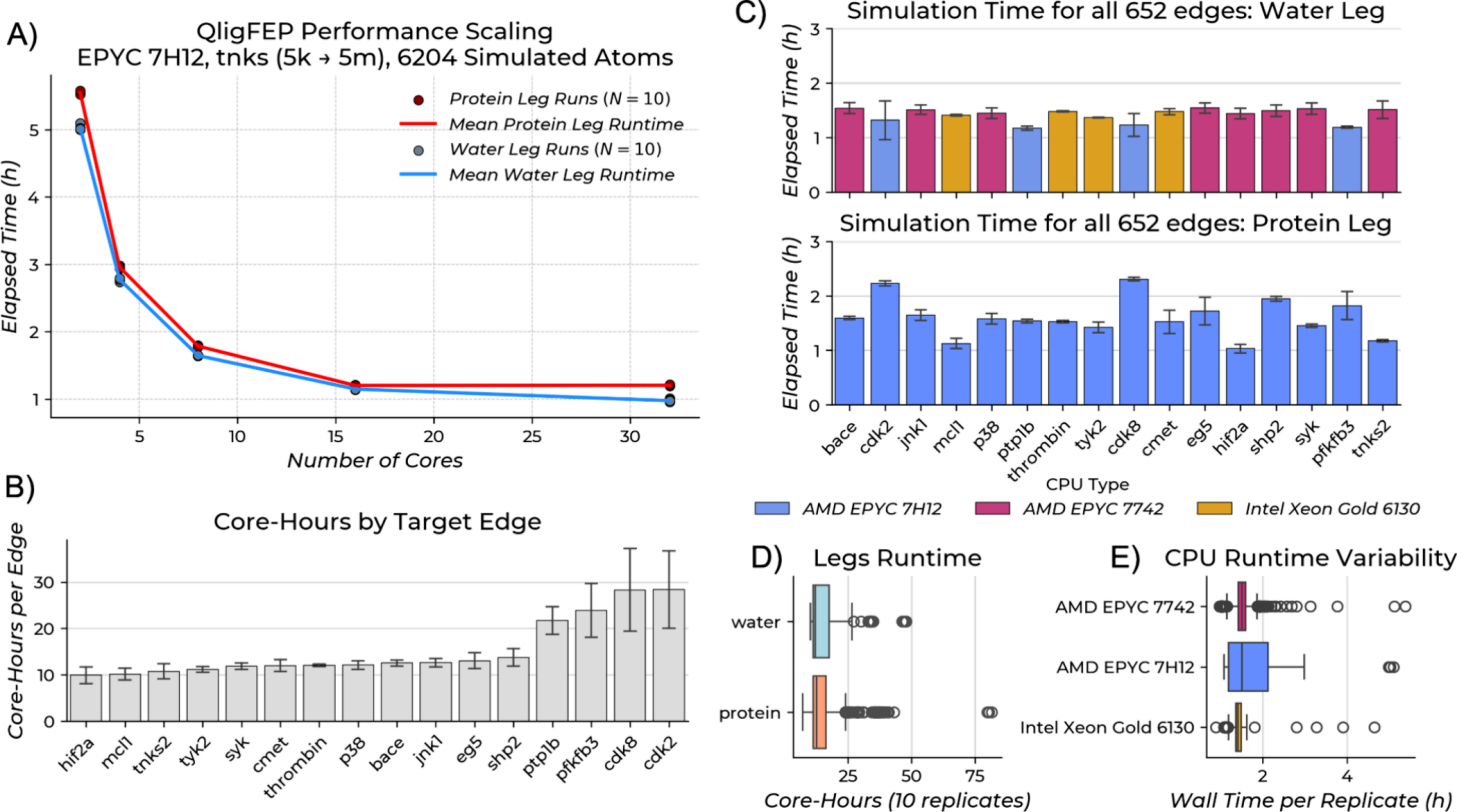
Computational scaling of QligFEP protocol and compute cost profile of the benchmarking experiments. (A) Parallel scaling performance of QligFEP simulations on AMD EPYC 7H12 processors using 2, 4, 8, 16, and 32 cores. The benchmark system consisted of the TNKS RBFE edge connecting ligands 5k and 5m (end-state) with 6,204 simulated atoms. Individual data points represent execution times for 10 independent replicates of both protein leg (dark red circles) and water leg (gray circles) free energy calculations. Solid lines show mean runtime for protein leg (red) and water leg (blue) simulations. Error bars represent standard deviation across replicates. (B) Mean core-hours required per edge across all protein targets, with error bars indicating the standard deviation. (C) Mean simulation time single FEP replicate for all benchmarked targets, with error bars indicating standard deviation. Bars are color-coded by the CPU architecture of the HPC systems employed. (D) Comparison of runtimes for protein and water legs of the thermodynamic cycle. (E) Variability in wall time per replicate for all different CPU architectures used by the HPC systems used for RBFE calculation, highlighting the broader spread for runs on AMD EPYC 7H12, associated with the I/O bottleneck of that HPC system.

The SBC approach simulates 6069 ± 92 (mean ± standard deviation) atoms for the protein leg and 6359 ± 9 for the water leg of the thermodynamic cycle (further detailed on Figure S4), dramatically smaller than full-system periodic boundary condition (PBC) simulations. Despite smaller system size, simulations capture essential protein–ligand interactions. Water legs generally require less time (13.78 ± 3.71 core-hours) than protein legs (16.53 ± 9.33 core-hours). Workload distributed across three HPC architectures (AMD EPYC 7H12, AMD EPYC 7742, Intel Xeon Gold 6130) showed consistent performance (1.38–1.60 h per replicate), demonstrating portability. Compute costs ranged from 9.91 core-hours per edge (hif2a) to 28.36 core-hours (cdk2; Figure 2B). Observed variability can be attributed to a documented filesystem issue on the AMD EPYC 7H12 system’s storage affecting applications with frequent I/O-intensive operations during benchmarking. Targets showing highest costs also exhibited largest standard deviations in completion times, indicating the elevated values likely reflect system-specific bottlenecks rather than true computational requirements. Overall, mean compute cost across all targets was 29.79 ± 3.79 h wall-clock time per edge (including 10 replicates for the protein and water system).

### Accuracy Compared to Established Methods

Having established computational efficiency, we evaluated accuracy against established FEP methods. Statistical comparison of absolute ΔΔ*G* prediction errors across all 16 targets (Mann–Whitney *U* tests with Holm correction; Figure 3) showed QligFEP exhibited no significantly greater deviations compared to PMX-Sage 2.0 (*p* > 0.05 after correction). Compared to FEP+ OPLS3e, QligFEP showed significantly larger deviations only for *shp2*, indicating SBC simulations introduce no systematic artifacts and remain competitive with commercial and open-source protocols. For pfkfb3, despite outliers, QligFEP deviations did not reach significance versus FEP+ OPLS3e, whereas PMX-Sage 2.0 did, placing QligFEP’s performance between the two methods.

**Figure 3:**
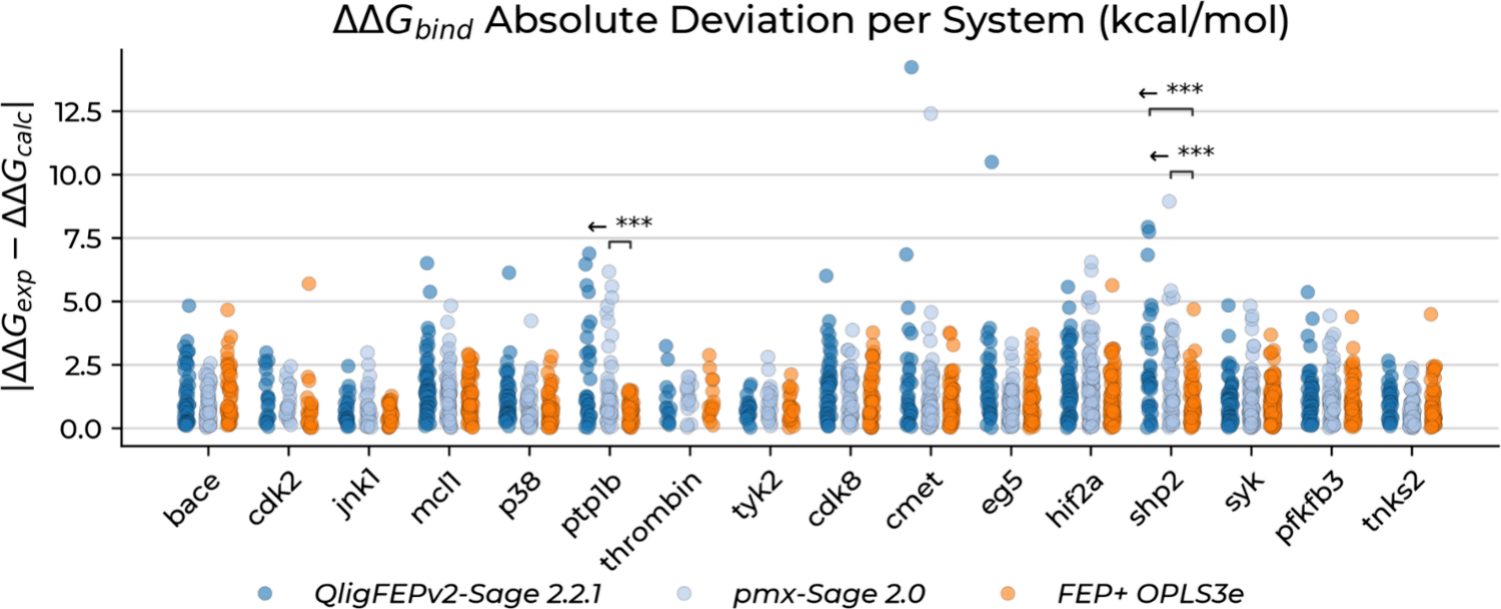
Absolute deviations between calculated ΔΔ*G* values (ΔΔ*G*_calc_) and experimental binding affinities (ΔΔ*G*_exp_) across all protein systems. Statistical tests were conducted using two directional Mann–Whitney *U* tests with the Holm correction method for multiple comparisons. Nonsignificant comparisons are omitted, while significant differences are shown as the noncorrected *p*-values and indicated by asterisks: *p < 0.05, **p < 0.01, ***p < 0.001, ****p < 0.0001. Arrows point toward the distribution with significantly higher values, indicated by the direction of the statistical test. Full test results are reported in Table S1.

The comparison of ranking performance (Kendall’s τ) and accuracy (MUE) across systems (Figure 4, Table 1) revealed target-dependent variations across all methods. Some targets show consistently strong performance (e.g., *p38*: τ > 0.58 all methods), while others present challenges (e.g., *mcl1*: τ < 0.25 all methods). Importantly, these variations are comparable across all three methods.

**Figure 4:**
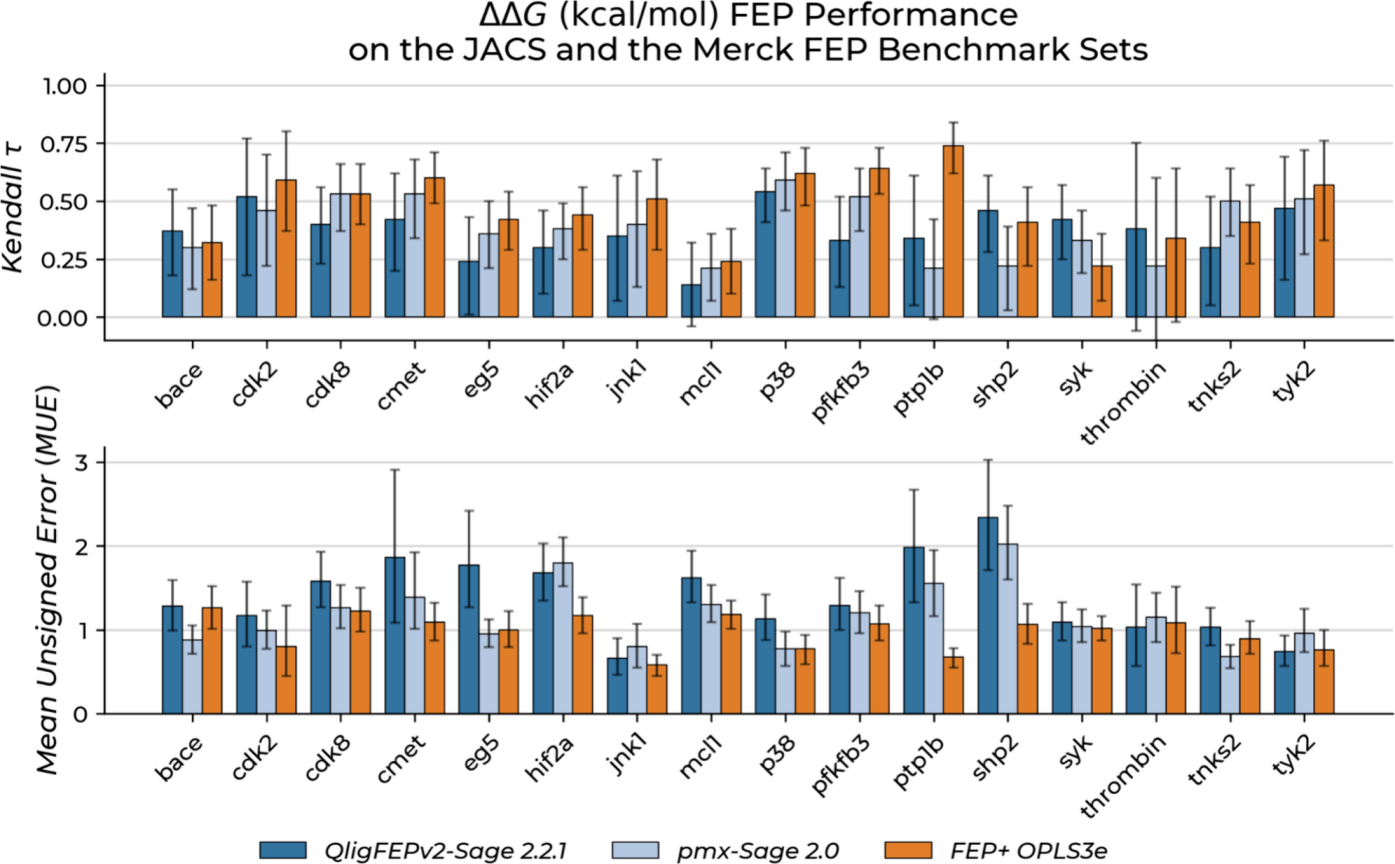
RBFE predictive performance of QligFEP compared to PMX-Sage 2.0 and FEP+ OPLS3e. On the top, Kendall’s τ is reported for all 16 investigated targets. On the bottom, the MUE. Error bars indicate 95% confidence intervals calculated using 1000 bootstrapped samples. Complete data, including Kendall’s τ, MUE, and number of edges per target are provided in Table 1.

**Table 1:** RBFE Predictive Performance of QligFEP Compared to pmx-Sage 2.0 (Denoted as pmx) and FEP+ OPLS3e (Denoted as OPLS3e) across 16 Targets^a^

	Kendall Tau			MUE			edge count		
target	OPLS3e	pmx	QligFEP	OPLS3e	pmx	QligFEP	OPLS3e	pmx	QligFEP
*bace*	0.32_0.16_^0.48^	0.3_0.12_^0.47^	0.37_0.18_^0.55^	1.26_1.01_^1.52^	0.88_0.71_^1.05^	1.28_0.99_^1.59^	58	58	49
*cdk2*	0.59_0.37_^0.8^	0.46_0.22_^0.7^	0.52_0.18_^0.77^	0.8_0.45_^1.29^	0.99_0.77_^1.23^	1.17_0.8_^1.57^	25	25	22
*cdk8*	0.53_0.4_^0.66^	0.53_0.37_^0.66^	0.4_0.23_^0.56^	1.22_0.98_^1.5^	1.26_1.02_^1.53^	1.58_1.27_^1.93^	54	54	55
*cmet*	0.6_0.49_^0.71^	0.53_0.34_^0.68^	0.42_0.2_^0.62^	1.09_0.87_^1.32^	1.39_1.01_^1.92^	1.86_1.08_^2.91^	57	57	34
*eg5*	0.42_0.29_^0.54^	0.36_0.21_^0.5^	0.24_0.01_^0.43^	1.0_0.79_^1.22^	0.95_0.79_^1.12^	1.77_1.27_^2.42^	65	65	38
*hif2a*	0.44_0.29_^0.56^	0.38_0.25_^0.49^	0.3_0.1_^0.46^	1.17_0.96_^1.39^	1.8_1.52_^2.1^	1.68_1.35_^2.03^	80	92	57
*jnk1*	0.51_0.29_^0.68^	0.4_0.13_^0.63^	0.35_0.07_^0.61^	0.58_0.45_^0.7^	0.8_0.55_^1.07^	0.66_0.46_^0.9^	31	31	27
*mcl1*	0.24_0.1_^0.38^	0.21_0.07_^0.36^	0.14_–0.04_^0.32^	1.18_1.01_^1.35^	1.3_1.09_^1.53^	1.62_1.33_^1.94^	71	71	60
*p38*	0.62_0.48_^0.73^	0.59_0.46_^0.71^	0.54_0.41_^0.64^	0.77_0.59_^0.94^	0.77_0.57_^0.98^	1.13_0.88_^1.42^	56	56	51
*pfkfb3*	0.64_0.53_^0.73^	0.52_0.37_^0.64^	0.33_0.13_^0.52^	1.07_0.87_^1.29^	1.2_0.96_^1.46^	1.29_1.0_^1.62^	66	66	48
*ptp1b*	0.74_0.62_^0.84^	0.21_–0.01_^0.42^	0.34_0.05_^0.61^	0.67_0.55_^0.78^	1.55_1.16_^1.95^	1.98_1.33_^2.67^	49	49	33
*shp2*	0.41_0.22_^0.56^	0.22_0.03_^0.39^	0.46_0.28_^0.61^	1.06_0.83_^1.31^	2.02_1.6_^2.48^	2.34_1.71_^3.03^	56	56	37
*syk*	0.22_0.07_^0.36^	0.33_0.19_^0.46^	0.42_0.25_^0.57^	1.02_0.87_^1.16^	1.04_0.85_^1.24^	1.09_0.87_^1.33^	101	101	59
*thrombin*	0.34_–0.02_^0.64^	0.22_–0.23_^0.6^	0.38_–0.06_^0.75^	1.08_0.72_^1.51^	1.15_0.85_^1.44^	1.03_0.57_^1.54^	16	16	14
*tnks2*	0.41_0.23_^0.57^	0.5_0.35_^0.64^	0.3_0.05_^0.52^	0.89_0.71_^1.1^	0.68_0.54_^0.82^	1.03_0.81_^1.26^	60	60	33
*tyk2*	0.57_0.33_^0.76^	0.51_0.27_^0.72^	0.47_0.16_^0.69^	0.76_0.57_^1.0^	0.96_0.73_^1.25^	0.74_0.57_^0.93^	24	24	22

a Values show Kendall’s τ, and mean unsigned error (MUE) with 95% confidence intervals represented as superscript and subscript numbers, respectively, calculated using 1000 bootstrapped samples. Edge count represents the number of alchemical perturbations performed for each target.

QligFEP demonstrated superior ranking versus PMX-Sage 2.0 for 6/16 targets, with substantial improvements for *shp2* (τ = 0.46 vs 0.22), *pfkfb3* (τ = 0.34 vs 0.21), and *syk* (τ = 0.42 vs 0.33). Modest improvements were observed for *bace*, *cdk2*, and *thrombin*. Conversely, QligFEP showed lower performance for *eg5*, *jnk1*, *cmet*, *cdk8*, and *pfkfb3*, with *pfkfb3* and *cdk8* showing the largest gaps.

Compared to FEP+ OPLS3e, the commercial method demonstrated advantages for most targets, particularly *pfkfb3* (τ = 0.74 vs 0.34) and *pfkfb3* (τ = 0.64 vs 0.33). QligFEP showed comparable or slightly better performance for *bace*, *shp2*, *syk*, and *thrombin.*

QligFEP MUE values were generally higher than the other methods for several targets (*bace*, *cmet*, *eg5*, *p38*), with little confidence interval overlap. FEP+ OPLS3e showed clear MUE advantages for *hif2a*, *pfkfb3*, and *shp2*, potentially reflecting proprietary force field benefits. Overall despite higher MUE values for some targets, QligFEP maintains ranking performance comparable to other methods. This is particularly relevant for lead optimization applications, where accurate ranking of congeneric compounds is typically more important than minimizing absolute prediction errors.^37^ Coupled with its lower computational cost, QligFEP becomes a practical option for large-scale FEP experiments. In cases with lower performance for compound affinity ranking, such as *cdk8* (τ = 0.40), cycle-closure correction rescued the ranking performance. Absolute binding free energy (Δ*G*) values showed τ = 0.57 for QligFEP, comparable to OPLS3e (τ = 0.57) and PMX-Sage 2.0 (τ = 0.60), indicating that despite higher edge-wise MUE values, our method still benefits from network-based error cancellation (Figure S5, Table S2).

### Handling Complex Chemical Transformations

A key capability of QligFEP’s dual topology implementation is handling substantial structural changes. Figure 5 illustrates a complex *syk* transformation between ligands CHEMBL3265020 (R = indole) and CHEMBL3264999 [R = N-(4-carbamoylphenyl)-benzamide]. Despite drastically different substituents with different size and flexibility, the calculation achieved reasonable experimental agreement (ΔΔ*G*_exp_ = −0.41, ΔΔ*G*_calc_ = −0.87 ± 0.40 kcal/mol). The RestraintSetter algorithm correctly identified that indole and phenyl moieties should remain unrestrained to avoid forcing unnatural interactions. This example demonstrates QligFEP’s ability to handle transformations challenging for single-topology methods.

**Figure 5:**
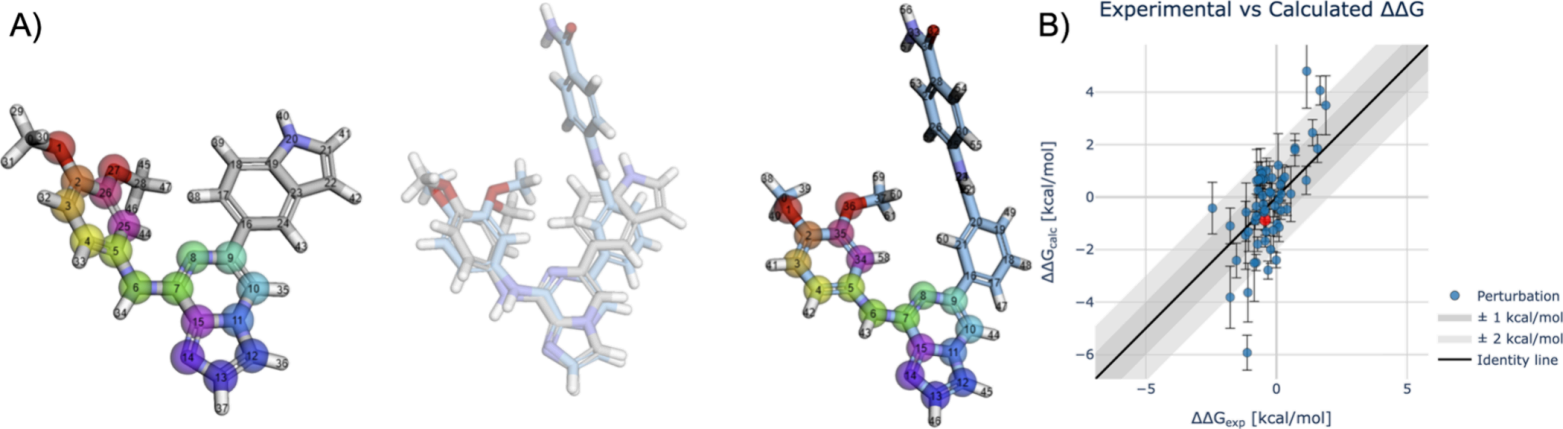
RBFE edge for *syk* involving ligands CHEMBL3265020 and CHEMBL3264999 (end-state). (A) Representation of the perturbed ligand (CHEMBL3265020) and its end-state. Colored spheres denote atom pairs connected by cross-topology distance restraints. Center: dual-topology representation showing both ligands superimposed in Cartesian space. Here, the para- and meta-substituted phenyl R-groups are likely to have different dynamics due to the bulkier substitution on the end-state ligand. Atom-atom distances from the phenyl rings yield no positional restraints on these groups, but a similar effect could be passed to the RestraintSetter algorithm otherwise. (B) Regression plot showing the experimental (ΔΔ*G*_exp_) by calculated binding affinities (ΔΔ*G*_calc_) in kcal/mol. The RBFE edge illustrated in (A) is highlighted in red, with ΔΔ*G*_calc_ = −0.87 ± 0.40 (SEM).

The automated RestraintSetter (Figure 6) provides flexible configurations: permissive (_p), less strict (_ls), and strict (_s) comparisons. Permissive heavy-atom comparisons (no R-group consideration) proved generally sensible across most networks. Stricter configurations may benefit scaffold hopping with R-groups having different flexibility and interaction profiles. Users should consider restraint appropriateness for their ligand series, adjusting based on structural similarity and degrees of freedom.

**Figure 6:**
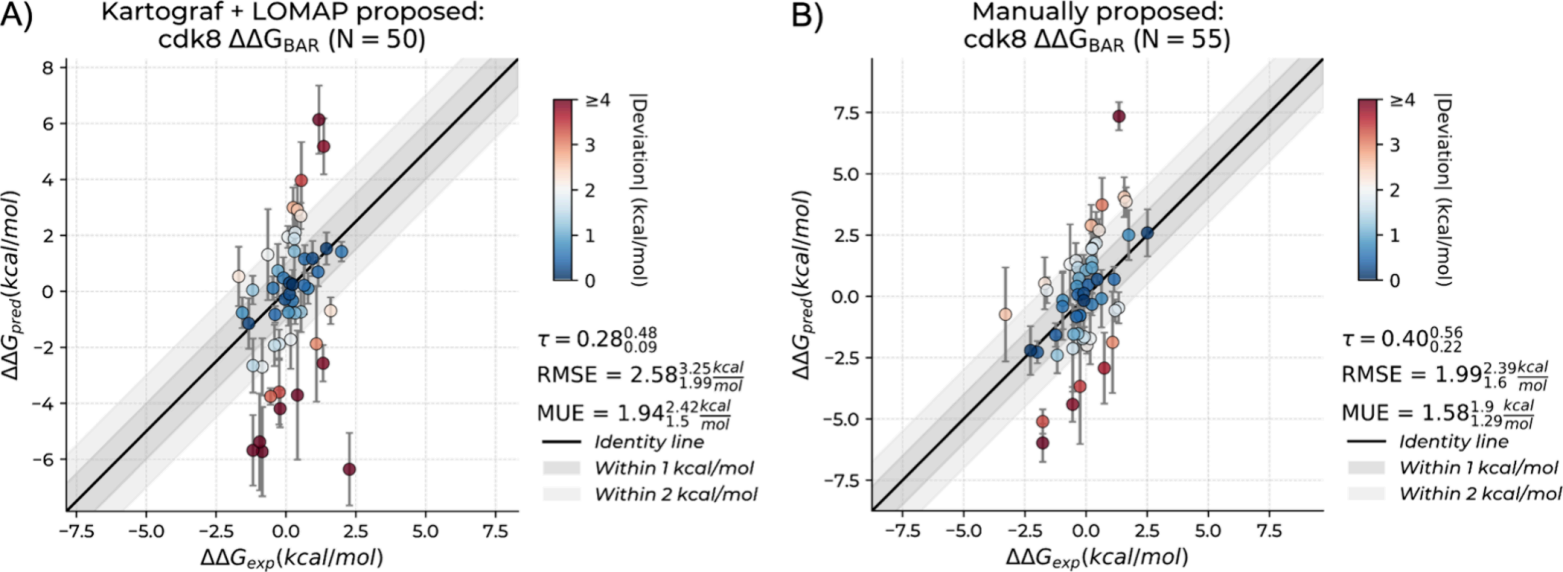
Performance comparison of automatically generated (left) versus manually designed (right) perturbation networks for *cdk8*. Plots represent the experimental (ΔΔ*G*_exp_) by calculated binding affinities (ΔΔ*G*_calc_) in kcal/mol. Kendall’s τ, RMSE and MUE are included with their respective 95% CI intervals calculated with bootstrapping, where the higher and lower bounds are reported as super and subscripts, respectively. Points are colored by absolute deviation from experimental values, with dark red points highlighted with absolute deviations of 4 or more kcal/mol. Error bars represent standard error of the mean. (A) Results from the initial automatic network. (B) Improved performance following manual network redesign.

### Importance of Input Structure Quality

Structure quality critically impacts FEP performance, as is well-established in the field. Ligand alignment and network design require careful attention regardless of input source, as inadequate alignment affects individual perturbation. Furthermore, when using Kartograf as a solution for atom mapping creation, suboptimal alignment may affect overall network topology. When ligands sharing large maximum common substructures (MCS) exhibited suboptimal structural overlap, we applied MCS-based alignment.^38^ We identified 4 such ligands in the *ptp1b* system and refined their alignment to optimize network topology (Figure S7). For the *shp2* data set, an alignment issue was observed for the edge connecting SHP099-1-7 and ligand 5 (Figure S8). Despite chemical equivalence, the chloro-substituted phenyl groups lacked positional restraints due to their poor structural alignment in their relative binding orientations, causing the edge to be largely overpredicted due to the unrestrained moieties. MCS-based ligand similarity clustering provides a systematic approach to identify compounds requiring refined alignment, enabling optimal structural correspondence for FEP calculations. In addition to our observations, the importance of ligand alignment for the success of FEP campaigns was also highlighted by the OpenFE consortium benchmark,^39^ which identified poor ligand alignment as a primary source of error across multiple systems.

### Network Design Considerations

Beyond individual perturbation quality, overall network topology significantly influences performance. For *cdk8*, automatic network generation produced challenging perturbations frequently involving ring to nonring atoms and occasionally simultaneously perturbing two separate chemical moieties (Figure S3). Manual network redesign yielded substantial improvements (Figure 6): Kendall’s τ increased from 0.28 (95% CI [0.09, 0.48]) to 0.40 (95% CI [0.22, 0.56]), while mean unsigned error decreased from 1.94 (95% CI [1.5, 2.42]) to 1.58 (95% CI [1.29, 1.9]) kcal/mol. This demonstrates that thoughtful network design (i.e.: avoiding overly complex transformations) can rescue performance for challenging systems. In addition to the automation steps provided in QligFEP, our workflow is flexible and provides sufficient tooling for cases that require manual curation from the user, such as mapping network design. In the future, integrating RestraintSetter-derived similarity values into network generation algorithms could be an effective strategy to improve automatic network design for our application, reducing the need for manual intervention.

## Conclusions and Future Prospects

QligFEP delivers an efficient, open-source framework with a CLI-based workflow for relative binding free energy calculations. Comprehensive benchmarking across 16 protein targets and 639 ligand transformations demonstrated that QligFEP achieves competitive accuracy compared to both open-source (PMX-Sage 2.0) and commercial (FEP+ OPLS3e) alternatives while requiring lower computational resources. The SBC simulation strategy reduces system size to 6214 ± 159 atoms and enables efficient, parallelizable edge calculations on standard CPU-based clusters with consistent runtimes across several protein systems. Altogether, our findings support QligFEP as an accessible alternative to GPU-dependent full-system approaches. Statistical analyses confirm this focused simulation approach introduces no systematic artifacts, maintaining predictive reliability while delivering exceptional efficiency.

The combination of workflow reproducibility, accuracy, and computational accessibility positions QligFEP as a practical solution for accelerating compound optimization, particularly valuable for research groups with limited computational infrastructure. Future developments will focus on enhancing stability for challenging perturbations through hybrid topology approaches incorporating soft-core potentials, further improving restraint handling, network optimization, and automation. Additionally, a GPU and cloud-enabled implementation is currently under development. These enhancements will strengthen QligFEP’s role as a transparent, scalable platform for structure-based drug design.

## Supplementary Material



## Data Availability

QligFEP v2.1.0 is available at https://github.com/qusers/Q. All input structures, FEP submission commands, results, and analysis scripts and notebooks used to reproduce the figures in this manuscript are available at the https://github.com/qusers/qligfepv2-BenchmarkExperiments repository.

## Notes

Additional experimental details, protein and ligand input refinement procedures, supplementary figures, tables, and statistical analyses supporting the free energy calculations (PDF)

## Abbreviations

AM1-BCC: Austin Model 1 with Bond Charge Corrections
AWS: Amazon Web Services
BAR: Bennett’s acceptance ratio
CLI: command-line interface
CPU: central processing unit
FEP: free energy perturbation
FF: force field
FF14SB: Amber force field 14SB
GPU: graphics processing unit
GUI: graphical user interface
HPC: high-performance computing
I/O: input/output
LOMAP: Lead Optimization Mapper
MCS: maximum common substructure
MUE: mean unsigned error
OpenFF: open force field initiative
OPLS3: optimized potentials for liquid simulations 3
OPLS: optimized potentials for liquid simulations
Q: molecular dynamics engine for free energy calculations
RBFE: relative binding free energy
RMSE: root-mean-square error
SBC: spherical boundary condition
SFC: state function-based correction
PBC: periodic boundary condition
SCAAS: surface constrained all-atom solvent model
SEM: standard error of the mean
TIP3P: three-point transferable intermolecular potential
τ: Kendall’s rank correlation coefficient
