# Supplementary material for: Doing More with Less: Accurate and Scalable Ligand Free Energy Calculations by Focusing on the Binding Site
Source: J Chem Inf Model. 2026 Feb 13;66(6):3164–72. doi: 10.1021/acs.jcim.5c02932 (PMC13014454; doi:10.1021/acs.jcim.5c02932)
Supplement: Supplementary file 1 [file ci5c02932_si_001.pdf]

Supporting Information

Doing More with Less: Accurate and Scalable Ligand Free Energy Calculations by Focusing on the Binding Site

David Alencar Araripe[1,2,3]; Alejandro Díaz-Holguín[3], Antti Poso[4], Gerard J.P. van Westen[3], Johan Åqvist[3], Hugo Gutiérrez-de-Terán[3,5,6], Willem Jespers[1,2,6*]*

[1] Department of Medicinal Chemistry, Photopharmacology and Imaging, Groningen Research. Institute of Pharmacy (GRIP), Faculty of Science and Engineering, Antonius Deusinglaan 1, 9713 AV Groningen, The Netherlands

[2] Division of Medicinal Chemistry, Leiden Academic Centre for Drug Research, Leiden University, P.O. Box 9502, 2300 RA, Leiden, The Netherlands

[3] Department of Cell & Molecular Biology, Uppsala University, Biomedical Center, SE-75124 Uppsala, Sweden

[4] School of Pharmacy, University of Eastern Finland, P.O. BOX 1627, 70211 Kuopio, Finland

[5] Nanomaterials and Nanotechnology Research Center (CINN), CSIC-University of Oviedo-Principado de Asturias, and Health Research Institute of Asturias (ISPA), Av. del Hospital Universitario, s/n, ES-33011 Oviedo, Asturias, Spain

[6] MODSIM Pharma AI B.V., Industrieweg 9, 2254AE, Voorschoten, The Netherlands

*correspondence: w.jespers@rug.nl, h.g.teran@cinn.es

Supplementary Text S1. Protein and Ligand Input Refinement 3
bace....S3
cdk2....S3
jnk1....S4
mcl1....S4
p38....S4
ptp1b....S5
thrombin....S5
tyk2....S5
cdk8....S5
cmet....S6
eg5....S6
hif2a....S6
pfkfb3....S6
shp2....S7
syk....S7
tnks2....S8
Supplementary Figures....S8
Supplementary Tables....S15

Supplementary Text S1. Protein and Ligand Input Refinement

FEP calculations, including those performed with QligFEP, require high-quality input structures, where significant steric clashes can compromise simulation stability. Consequently, manual curation including minor adjustments to the input structures were required in a number of cases from the benchmark dataset to alleviate such clashes. Additionally, this curation stage included residue and atom naming modifications to the source PDB files to conform to specific conventions of our force field implementation. Structural manipulations including visual inspection, residue selection, and energy minimization, were performed using Schrödinger's Maestro.[23] Energy minimization steps were applied locally by selecting protein residues and by using Maestro's integrated tools, corresponding to a default minimization protocol such as steepest descent followed by L-BFGS with an OPLS-based force field to relieve clashes or optimize local geometry.

bace

Energy minimization was applied to binding pocket residues Ile171, Ser96, Ser71, Phe169, and Gly291 to resolve steric clashes using Maestro.

cdk2

Energy minimization was applied to binding pocket residues Lys89, Asp86, and Leu138 to resolve steric clashes using Maestro. Furthermore, an adjustment was made to the input ligand structures for the cdk2 series due to suboptimal correlation with experimental data when using the ligand

poses from the IndustryBenchmarks2024 repository. Ligand 17, featuring a meta-bromophenyl, initially adopted a solvent-exposed conformation. Guided by the crystallographic structure 6GUK featuring a similar halogenated moiety in a less solvent-exposed rotamer (Figure S1A), we reoriented the halogen in ligand 17 to adopt this alternative conformation (Figure S1B, S1C). While the electron density maps indicated some positional uncertainty for the halogen in 6GUK, this conformational adjustment for ligand 17 demonstrably improved the correlation between QligFEPv2 results and experimental binding affinities, supporting its use.

jnk1

Protein-ligand complexes were loaded into Maestro. Energy minimization was applied to residues Gly35, Val40, Leu110, Met111, and Ala113 to mitigate steric clashes and improve the accommodation of the congeneric ligand series.

mcl1

Upon loading the protein-ligand complexes into Maestro, energy minimization was applied to the binding site residues Val253, Met231, Leu246, Leu290, Ile294, Leu267, Met250, Val274, Leu235, Phe270, and Gly271 to optimize ligand interactions and prevent steric hindrance.

p38

No manual protein minimization was required for this system. Observed steric hindrance was limited to interactions with crystallographic water molecules, which are systematically removed by QligFEPv2 prior to Relative Binding Free Energy (RBFE) simulations.

ptp1b

An internally prepared ptp1b structure, previously validated for QligFEP RBFE calculations, was employed. An assessment of the ligand poses revealed suboptimal alignment of peripheral ligand regions, despite good Maximum Common Substructure (MCS) alignment of the core scaffold (Figure S6). An additional ligand alignment process was run to further refine the starting poses, which resulted in a better perturbation network based through improved Kartograf atom mappings. In addition, distance restraints applied to perturbations involving these ligands were consequently improved, a direct consequence of the improved cartesian overlap.

thrombin

The protein structure from the IndustryBenchmarks2024 repository presented issues by the time the input structures were extracted. These included suboptimal hydrogen positioning and incorrect amino acid sequencing. An internally prepared ptp1b structure, previously validated for QligFEP RBFE calculations, was employed.

tyk2

For the *tyk2* system, energy minimization was applied to binding pocket residues LEU903, Tyr980, Gly984, and Pro982 within Maestro to resolve steric clashes.

cdk8

Protein-ligand complexes were processed in Maestro. Energy minimization was applied to the residues Val27, Gly28, Tyr32, Lys52, Ile79, His102, Asp103, Asn156, Leu158, and Arg356 to prevent steric clashes. In addition, we observed the presence of multiple challenging perturbations in the generated map (Figure S3A), often involving the perturbation of non-aromatic ligand atoms to ring structures or the perturbation of two separate molecular moieties at once. To counteract this, we manually designed an alternative perturbation mapping, minimizing the amount of challenging perturbations for our method (Figure S3B), which was subsequently used to conduct the FEP experiments.

cmet

Binding site residues Ile1084, Gly1085, Met1160, and Lys1161 underwent energy minimization to resolve steric issues.

eg5

Energy minimization was applied to residues Arg119, Pro121, Leu160, Gly217, and Ala218 to address steric hindrance with the congeneric ligands.

hif2a

Energy minimization was applied to residues Met289, His293, and Cys339 to resolve steric clashes in the binding site.

pfkfb3

Energy minimizations applied to resolve steric clashes in the binding site were applied through two steps. Initially, ligands 20, 41, and 42 were included in the workspace, and an energy minimization was applied to the residue Val214. In sequence, ligands 44, 47, 52, and 53 were loaded in the workspace and an energy minimization was applied to residues Leu238, Ile241, and His242. Water molecules 905, 914, 944, 993, 998, and 1022 (residue HOH) were removed based on steric clashes with other water molecules and the lack of direct interactions with the protein residues.

In addition, an adjustment to ligand poses was implemented for the *pfkfb3* series upon observing a poor correlation of FEP edges involving ligand 43. We observed that the meta-bromophenyl rotamer of this ligand present in the benchmarking set was in the opposite orientation compared to other congeneric ligands (Figure S2B). The crystal structure of *pfkfb3* with ligand 38 (PDB ID

6HVI), a congener featuring a meta-substituted ring at corresponding coordinates, indicates an alternative rotamer position (Figure S2A). Therefore, upon observing the good overlap of the electron density and the cocrystallization ligand 38, we opted to reorient the meta-bromophenyl group to match ligand 38 (Figure S2C). This change resulted in an improved correlation between calculated and experimental binding affinities, further validating the decision.

shp2

Energy minimization was applied to residues Phe113, His114, Thr219, Glu249, Asp489, and Lys492 to prevent steric hindrance with the congeneric ligands.

syk

Energy minimization was applied to residues Glu376, Leu377, Gly378, Val385, Asn457, Asp512, Phe513, Lys402, Gly454, Ser379, Lys375, Phe382, and Lys458, to prevent steric hindrance with the congeneric ligands. Additional minimizations were performed on other protein regions to resolve inter-residue clashes within the protein structure. Finally, the protonation states and hydrogen bonding network were optimized using Maestro's Refine > H-bond-assignment tool, with options selected to sample water orientations and utilize PROPKA-predicted pKa values at pH 7.0.

tnks2

The tnks2 system, as obtained from the IndustryBenchmarks2024 repository, was utilized without further structural modifications, as it presented no immediate steric concerns or incompatibilities with our FEP workflow.

Supplementary Figures

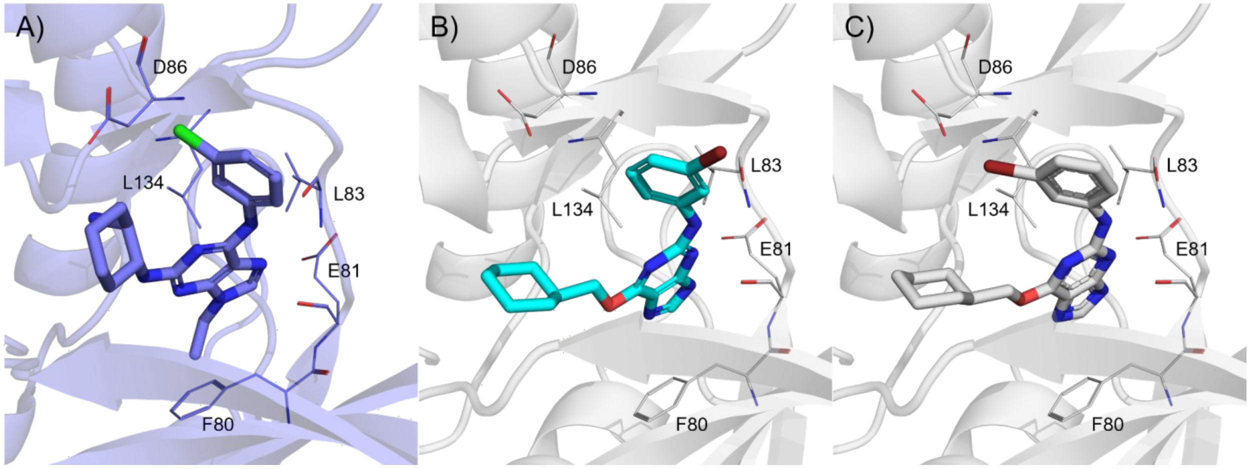

Figure S1. Ligand pose refinement for the benchmarked target *cdk2*. A) Crystal structure of *cdk2* (PDB ID: 6GUK) with a co-crystallized ligand structurally similar to ligand 17 from the *cdk2* JACS benchmark set. B) *cdk2* protein pose (white) and ligand 17 (cyan) as obtained from the IndustryBenchmarks2024 repository, which provides the starting benchmarking ligand and protein poses. C) *cdk2* protein pose (white) and the modified rotamer of ligand 17 (white), in which the meta-bromophenyl group is flipped to match the ligand orientation observed in 6GUK (A).

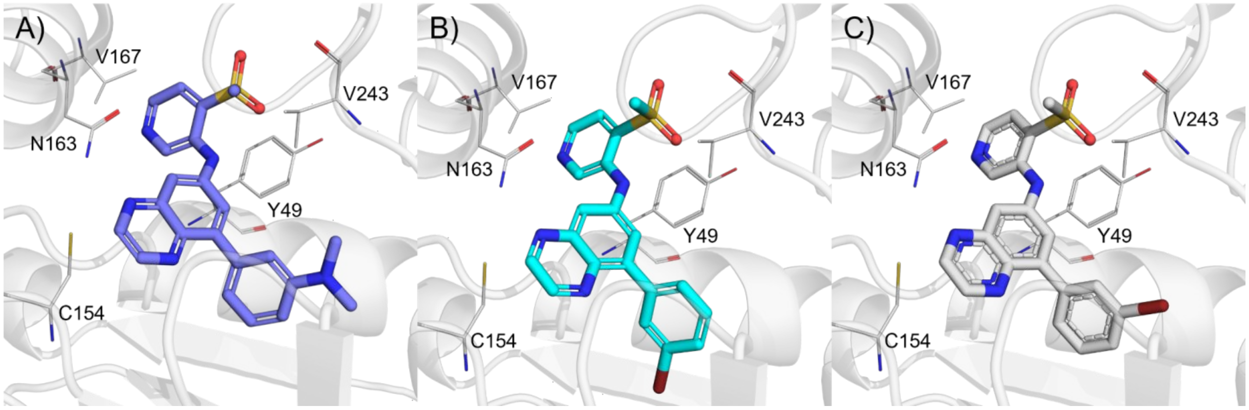

Figure S2. Ligand pose refinement for the benchmarked target *pfkfb3* (white). A) Co-crystallized ligand in *pfkfb3* (PDB ID: 6HVI; ligand 38 in the series). B) Ligand 43, as obtained from the source repository containing starting ligand and protein poses (IndustryBenchmarks2024). C) Modified rotamer of ligand 43 in which the meta-bromophenyl group is flipped to match the orientation of the co-crystallized ligand shown in (A).

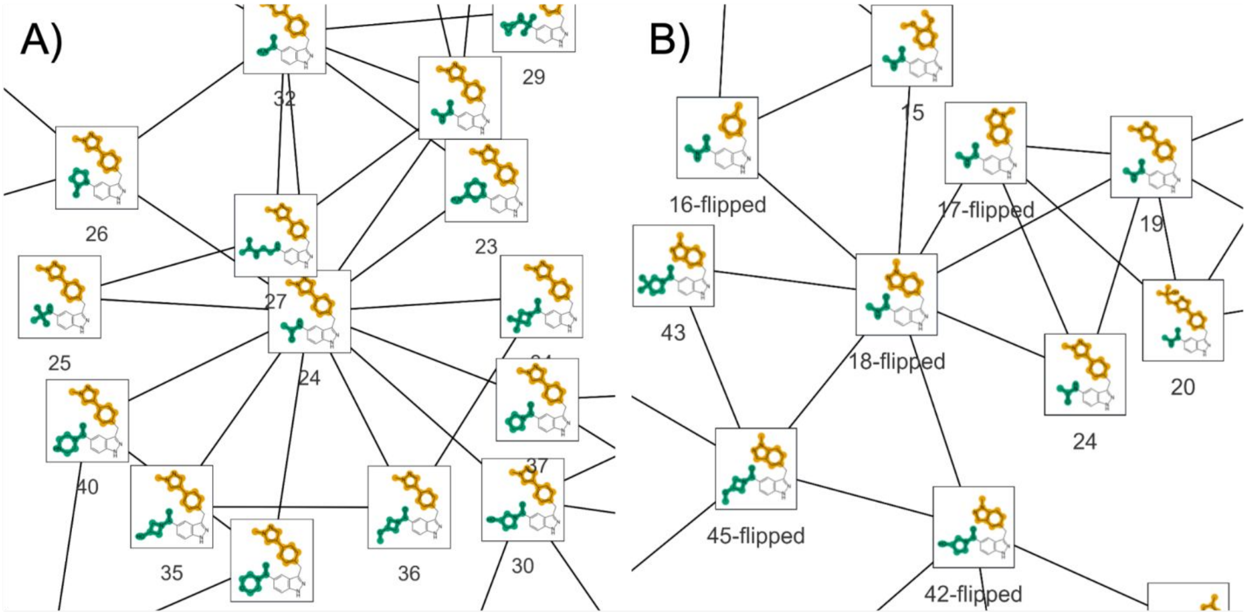

Figure S3. *cdk8* perturbation mapping comparison. A) Automatic mapping obtained with the Kartograf atom mapper and the LOMAP perturbation scoring method. The two moieties perturbed across all the edges are highlighted in green and yellow. At the center, ligand 24 is perturbed to other ligands, often involving large perturbations with the introduction of ring atoms. B) The manual mapping used in our benchmarking. Ligand 24 is displayed connected to ligands 17, 18, and 19, representing better perturbation edges for our dual topology approach and restraint method, which by default do not restrain ring to non-ring atoms.

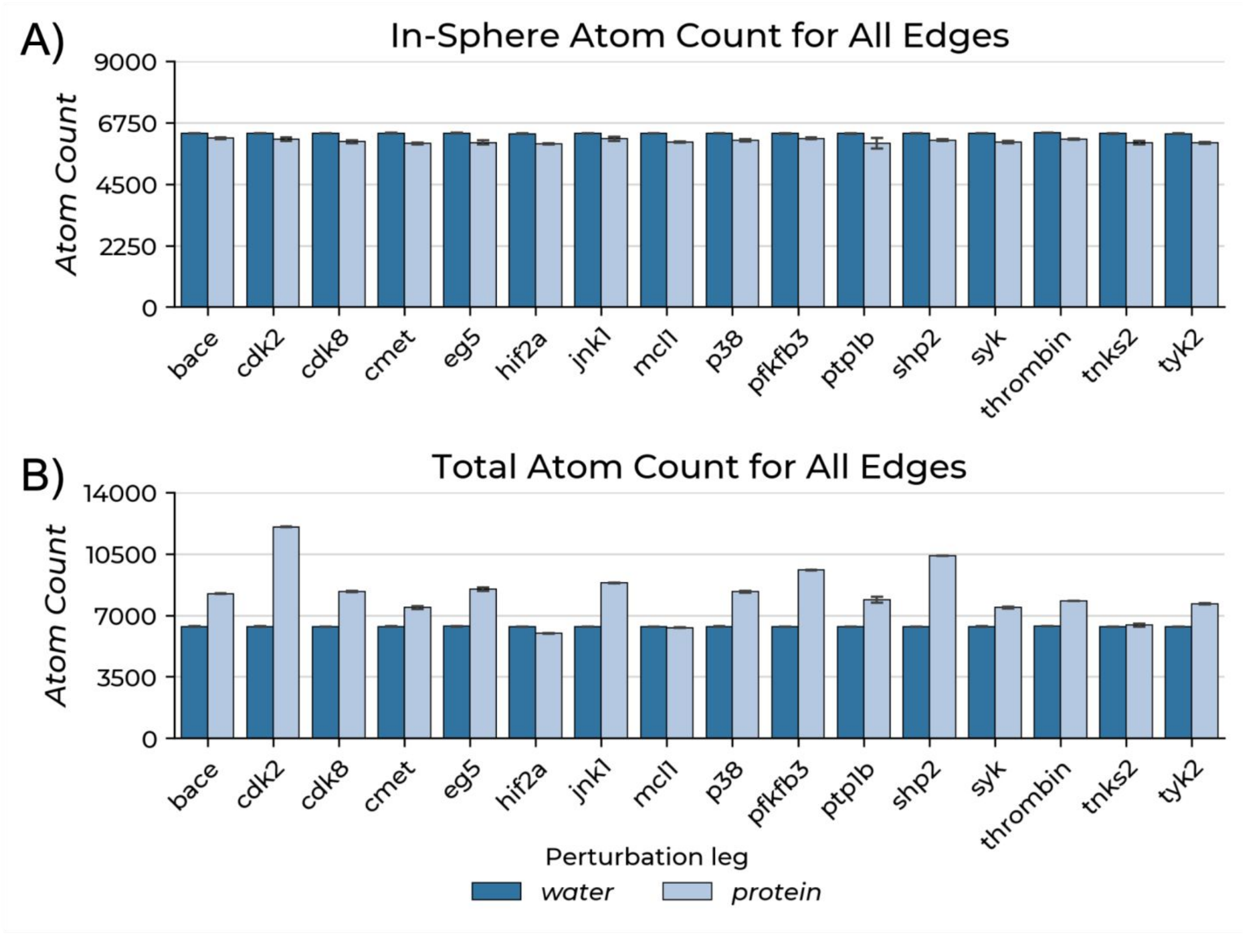

Figure S4. Number of simulated atoms in all perturbation edges, including dual ligand topologies, per protein target. The different perturbation legs, protein and water, are denoted in different colors, with error bars denoting the standard deviation of the atom count for all simulated alchemical transformations. A) In-sphere atom count per target and perturbation leg. B) Total atom count per target and perturbation leg. Computational overhead is introduced with out-of-sphere atoms, as bonded terms are still calculated during the simulation time.

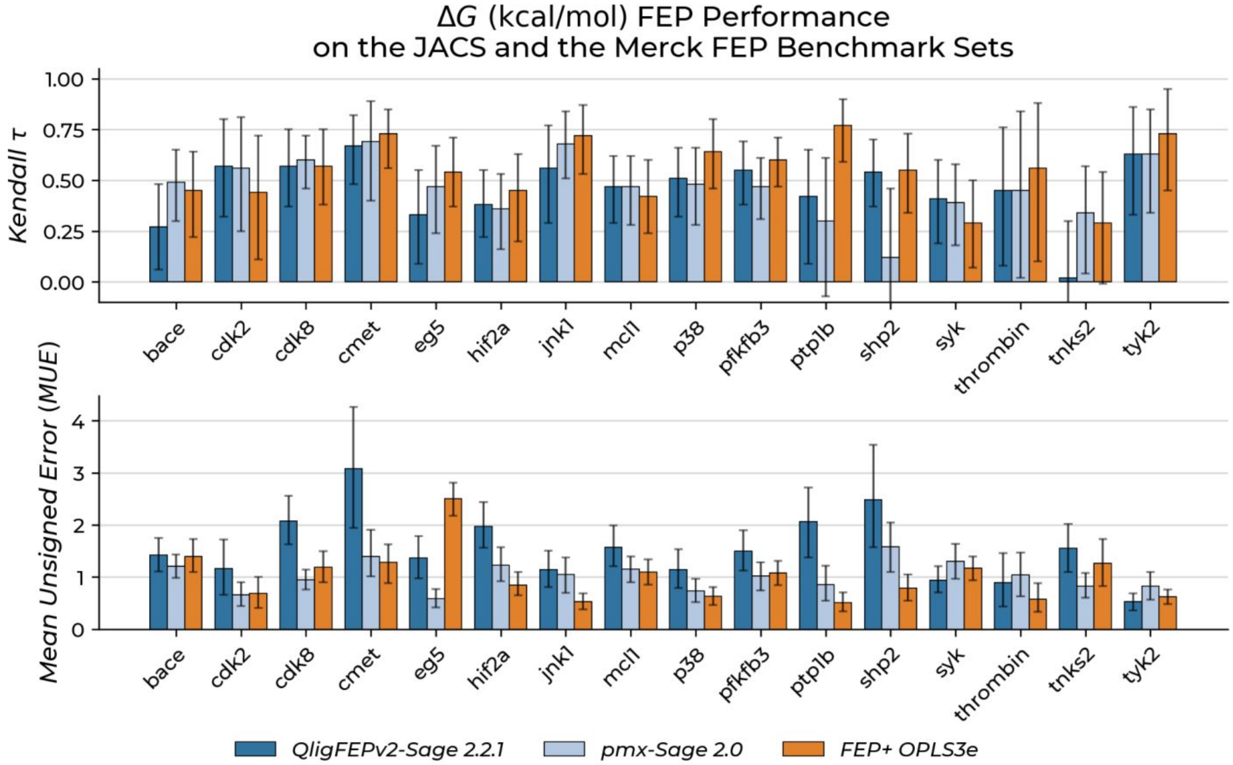

Figure S5. Cycle closure-corrected binding affinity (ΔG_{bind}) estimation performance of QligFEP compared to PMX-Sage 2.0 and FEP+ OPLS3e. On the top, Kendall's τ is reported for all 16 investigated targets. On the bottom, the MUE. Error bars indicate 95% confidence intervals

calculated using 1000 bootstrapped samples. Complete data, including Kendall's τ, MUE, and number of ligands per target are provided in Table S2.

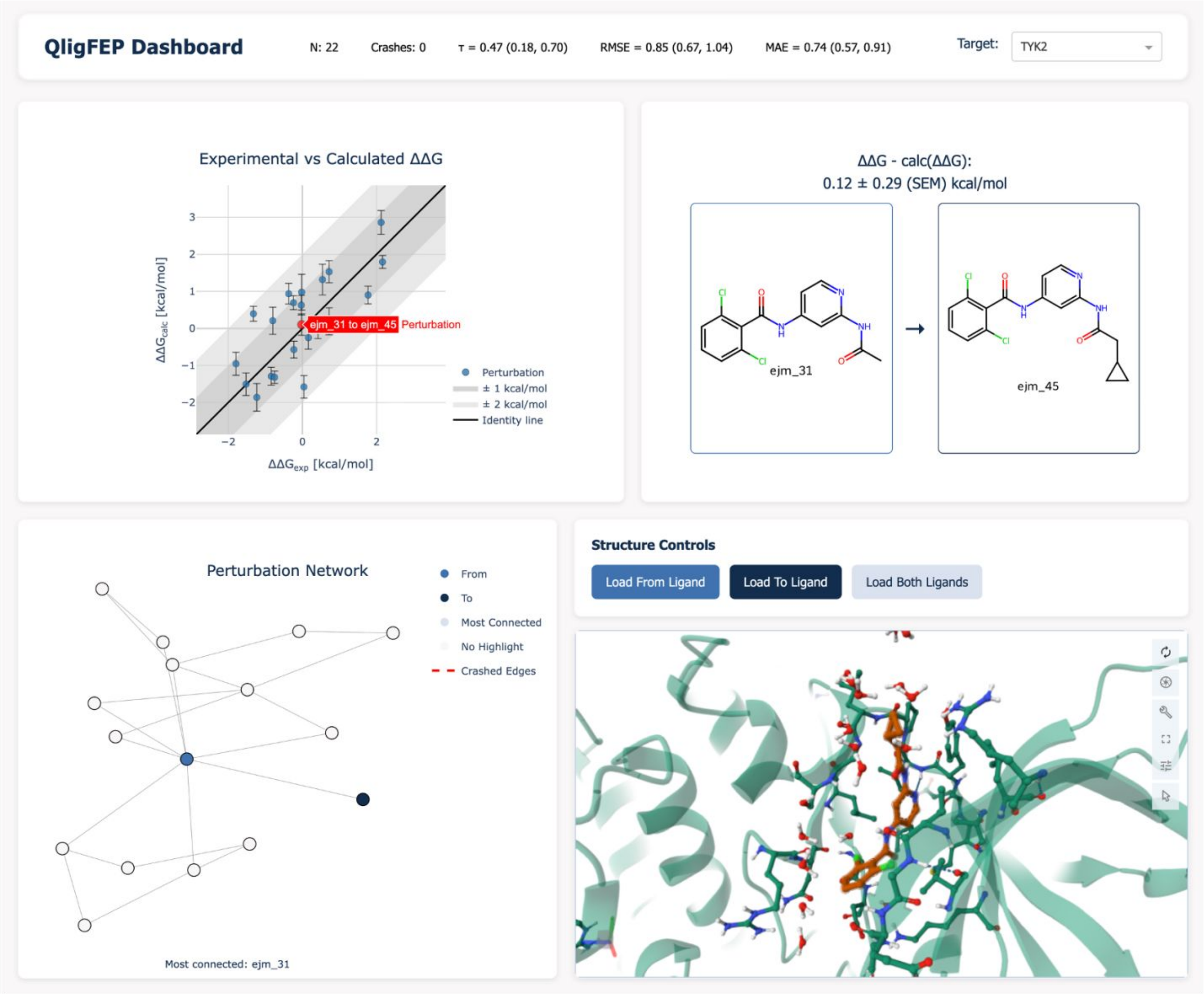

Figure S6. QligFEP dashboard. On the top, the perturbation status is displayed, containing the number of edges ran for a system (N), the number of crashed edges, the Kendall τ, RMSE and MAE of the whole perturbation run; with lower and upper bounds of the bootstrapped metric displayed in parenthesis. On the right, a dropdown menu is available to change the target system

displayed on the dashboard. In the figure, the dashboard displays results for the target *tyk2*. On the top left of the dashboard, a click-reactive regression plot of the results is displayed, representing the calculated per experimental $\Delta\Delta G_{bind}$ values. On the top right, a two-dimensional representation of the initial (ejm_31) and end state (ejm_45) ligands is shown. On the bottom left, the perturbation network is displayed, highlighting the most connected node and the ligands involved in the perturbation, with the initial and end states denoted as "from" and "to", respectively. On the bottom right, an interactive depiction of the protein, water droplet and ligands involved in the perturbation are rendered, powered by Mol*. In the image, the end-state ligand is displayed. On top of this panel, structure control buttons are displayed, allowing the user to load the protein structure with the initial state "from", the end state "to", or both ligand states at once, fully representing our dual topology approach.

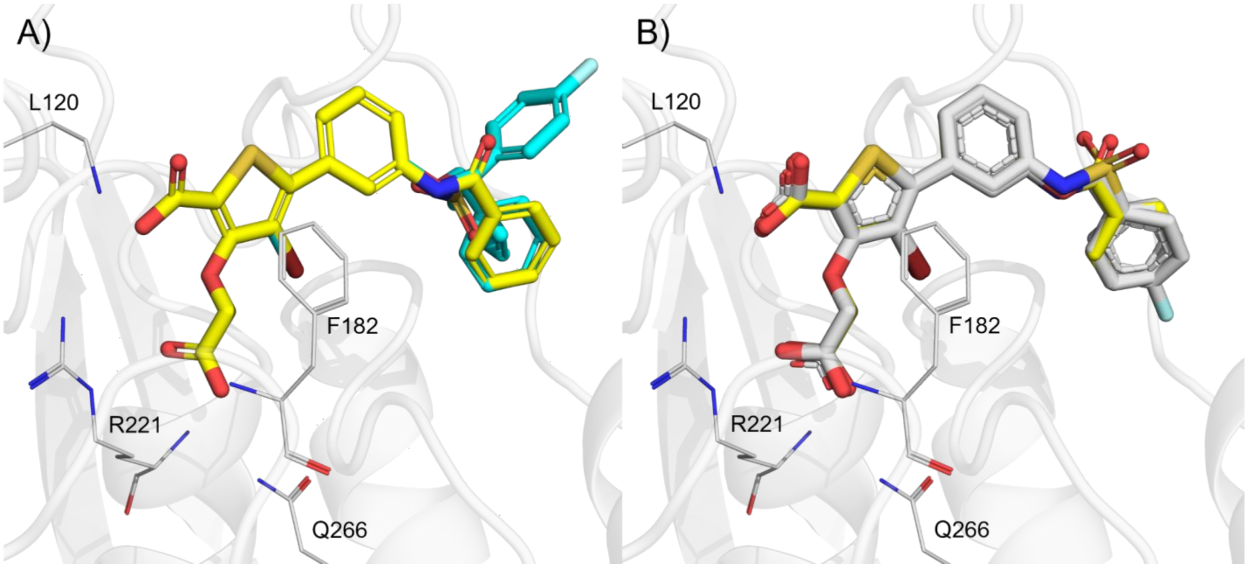

Figure S7. Ligand pose refinement for the benchmarked target *ptp1b*. A) *ptp1b* protein pose used in this benchmarking study (white) and ligands 23469 (yellow), 23473, 20669, and 23472 (cyan)

obtained from the source repository containing starting ligand and protein poses (IndustryBenchmarks2024). B) Upon aligning ligands 23473, 20669, and 23472 to reference ligand 23469 (yellow), we obtained the final poses used in this benchmarking study (white).

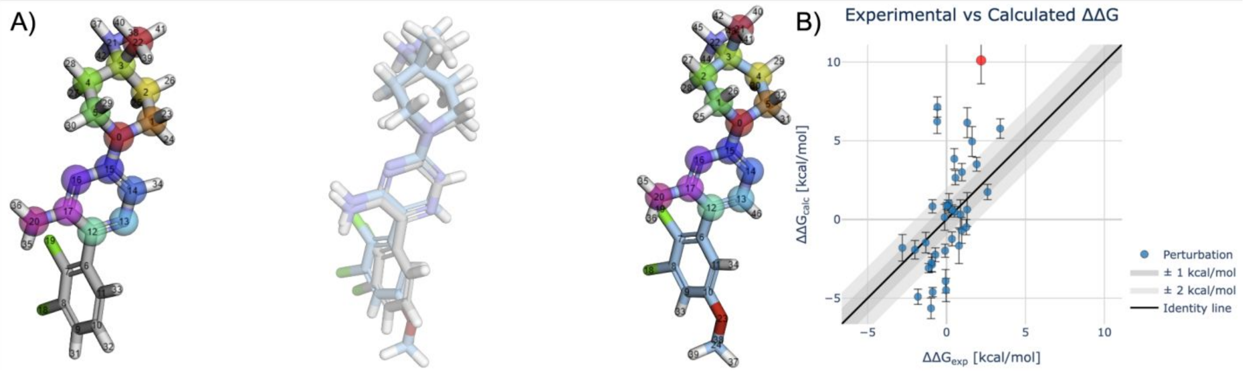

Figure S8. Outlier RBFE edge for *shp2*, involving SHP099-1-7 and ligand 5 (end-state). Representation of the perturbed ligand (SHP099-1-7) and its end-state. Colored spheres denote atom pairs connected by cross-topology distance restraints. Center: dual-topology representation showing both ligands superimposed in Cartesian space. Here, the chloro-substituted phenyl ring displays poor overlay across ligand topologies, causing the atom mapping not to be registered by Kartograf despite the increased atom maximum distance parameter, causing large perturbation error. B) Regression plot showing the experimental ($\Delta\Delta G_{exp}$) by calculated binding affinities ($\Delta\Delta G_{calc}$) in kcal/mol. The RBFE edge illustrated in (A) is highlighted in red, with $\Delta\Delta G_{calc}$ = 10.11 ± 1.49 (SEM).

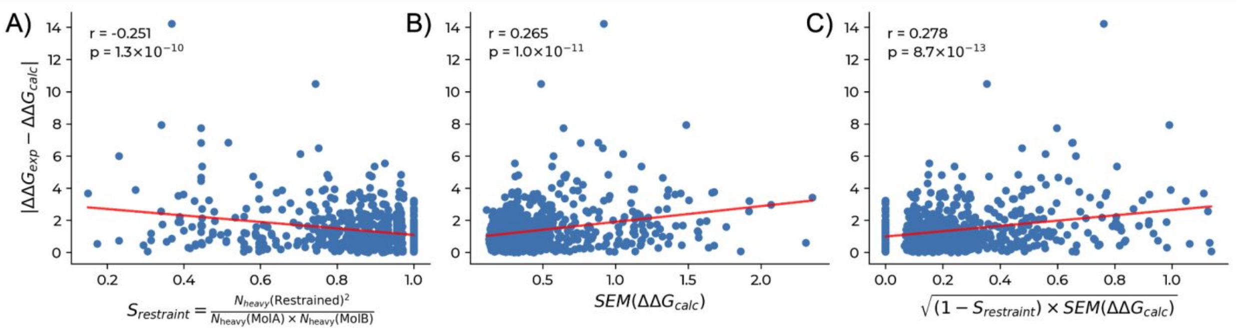

Figure S9. Pearson correlation between ad hoc scores and absolute deviations from experimental binding affinities. Three scoring metrics are evaluated: A) Restraint similarity, calculated as the heavy atom Johnson similarity between the restrained moiety and the heavy atoms in MolA and MolB; B) standard error of the mean (SEM) of calculated $\Delta\Delta G_{bind}$ values, propagated from protein and water legs of the thermodynamic cycle; and C) combined score, defined as the geometric mean of restraint distance (1 - similarity) and SEM. Red lines represent linear regression fits to the data points, with the Pearson coefficient (r) and the respective *p*-value reported on the top left of the plots.

Supplementary Tables

Table S1. Statistical testing results for force field comparisons in Figure 2. Each row represents a pairwise comparison between force fields for a specific protein system, showing the two groups being compared (with sample sizes N), the original (uncorrected) *p*-value from the Mann–Whitney U test, the corrected *p*-value after Holm correction for multiple comparisons, and the test direction.

Only comparisons with corrected p-values < 0.05 are shown in Figure 2, with statistical significance displayed as uncorrected p-values: *$p < 0.05$, **$p < 0.01$, ***$p < 0.001$, ****$p < 0.0001$. For these significant comparisons, test direction indicates which group has higher absolute deviations (i.e., worse performance): "Group 1 > Group 2" means Group 1 performs worse, "Group 2 > Group 1" means Group 2 performs worse.

Group 1	Group 2	Original p-value	Corrected p-value	Test Direction
cdk2 QligFEPv2-Sage 2.2.1, ($N = 22$)	cdk2 pmx-Sage 2.0, ($N = 25$)	0.461784	1	Group 1 > Group 2
cdk2 pmx-Sage 2.0, ($N = 25$)	cdk2 FEP+ OPLS3e, ($N = 25$)	0.0239029	0.874041	Group 1 > Group 2
jnk1 QligFEPv2-Sage 2.2.1, ($N = 27$)	jnk1 pmx-Sage 2.0, ($N = 31$)	0.657371	1	Group 1 > Group 2
jnk1 pmx-Sage 2.0, ($N = 31$)	jnk1 FEP+ OPLS3e, ($N = 31$)	0.306138	1	Group 1 > Group 2
mcl1 QligFEPv2-Sage 2.2.1, ($N = 60$)	mcl1 pmx-Sage 2.0, ($N = 71$)	0.0681379	1	Group 1 > Group 2
mcl1 pmx-Sage 2.0, ($N = 71$)	mcl1 FEP+ OPLS3e, ($N = 71$)	0.299331	1	Group 1 > Group 2
p38 QligFEPv2-Sage 2.2.1, ($N = 51$)	p38 pmx-Sage 2.0, ($N = 56$)	0.0035575	0.149415	Group 1 > Group 2
p38 pmx-Sage 2.0, ($N = 56$)	p38 FEP+ OPLS3e, ($N = 56$)	0.661322	1	Group 1 > Group 2
ptp1b QligFEPv2-Sage 2.2.1, ($N = 33$)	ptp1b pmx-Sage 2.0, ($N = 49$)	0.260115	1	Group 1 > Group 2
ptp1b pmx-Sage 2.0, ($N = 49$)	ptp1b FEP+ OPLS3e, ($N = 49$)	0.000823061	0.0378608	Group 1 > Group 2
thrombin QligFEPv2-Sage 2.2.1, ($N = 14$)	thrombin pmx-Sage 2.0, ($N = 16$)	0.835693	1	Group 1 > Group 2
thrombin pmx-Sage 2.0, ($N = 16$)	thrombin FEP+ OPLS3e, ($N = 16$)	0.208881	1	Group 1 > Group 2
tyk2 QligFEPv2-Sage 2.2.1, ($N = 22$)	tyk2 pmx-Sage 2.0, ($N = 24$)	0.851878	1	Group 1 > Group 2
tyk2 pmx-Sage 2.0, ($N = 24$)	tyk2 FEP+ OPLS3e, ($N = 24$)	0.139508	1	Group 1 > Group 2
cdk8 QligFEPv2-Sage 2.2.1, ($N = 55$)	cdk8 pmx-Sage 2.0, ($N = 54$)	0.108726	1	Group 1 > Group 2
bace QligFEPv2-Sage 2.2.1, ($N = 49$)	bace pmx-Sage 2.0, ($N = 58$)	0.0756447	1	Group 1 > Group 2
bace pmx-Sage 2.0, ($N = 58$)	bace FEP+ OPLS3e, ($N = 58$)	0.970699	1	Group 1 > Group 2
cdk8 pmx-Sage 2.0, ($N = 54$)	cdk8 FEP+ OPLS3e, ($N = 54$)	0.387547	1	Group 1 > Group 2
cmet QligFEPv2-Sage 2.2.1, ($N = 34$)	cmet pmx-Sage 2.0, ($N = 57$)	0.33633	1	Group 1 > Group 2
cmet pmx-Sage 2.0, ($N = 57$)	cmet FEP+ OPLS3e, ($N = 57$)	0.277801	1	Group 1 > Group 2
eg5 QligFEPv2-Sage 2.2.1, ($N = 38$)	eg5 pmx-Sage 2.0, ($N = 65$)	0.00211579	0.090979	Group 1 > Group 2
eg5 pmx-Sage 2.0, ($N = 65$)	eg5 FEP+ OPLS3e, ($N = 65$)	0.284203	1	Group 1 > Group 2
hif2a QligFEPv2-Sage 2.2.1, ($N = 57$)	hif2a pmx-Sage 2.0, ($N = 92$)	0.639598	1	Group 1 > Group 2
hif2a pmx-Sage 2.0, ($N = 92$)	hif2a FEP+ OPLS3e, ($N = 92$)	0.00356405	0.149415	Group 1 > Group 2
shp2 QligFEPv2-Sage 2.2.1, ($N = 37$)	shp2 pmx-Sage 2.0, ($N = 56$)	0.284652	1	Group 1 > Group 2
shp2 pmx-Sage 2.0, ($N = 56$)	shp2 FEP+ OPLS3e, ($N = 56$)	0.00056524	0.0265663	Group 1 > Group 2
syk QligFEPv2-Sage 2.2.1, ($N = 59$)	syk pmx-Sage 2.0, ($N = 101$)	0.204959	1	Group 1 > Group 2
syk pmx-Sage 2.0, ($N = 101$)	syk FEP+ OPLS3e, ($N = 101$)	0.73312	1	Group 1 > Group 2
pfkfb3 QligFEPv2-Sage 2.2.1, ($N = 48$)	pfkfb3 pmx-Sage 2.0, ($N = 66$)	0.366371	1	Group 1 > Group 2
pfkfb3 pmx-Sage 2.0, ($N = 66$)	pfkfb3 FEP+ OPLS3e, ($N = 66$)	0.216204	1	Group 1 > Group 2
tnks2 QligFEPv2-Sage 2.2.1, ($N = 33$)	tnks2 pmx-Sage 2.0, ($N = 60$)	0.00388605	0.155442	Group 1 > Group 2

Group 1	Group 2	Original p-value	Corrected p-value	Test Direction
tnks2 pmx-Sage 2.0, ($N = 60$)	tnks2 FEP+ OPLS3e, ($N = 60$)	0.891784	1	Group 1 > Group 2
cdk2 QligFEPv2-Sage 2.2.1, ($N = 22$)	cdk2 FEP+ OPLS3e, ($N = 25$)	0.0358084	1	Group 1 > Group 2
jnk1 QligFEPv2-Sage 2.2.1, ($N = 27$)	jnk1 FEP+ OPLS3e, ($N = 31$)	0.475141	1	Group 1 > Group 2
mcl1 QligFEPv2-Sage 2.2.1, ($N = 60$)	mcl1 FEP+ OPLS3e, ($N = 71$)	0.0236227	0.874041	Group 1 > Group 2
p38 QligFEPv2-Sage 2.2.1, ($N = 51$)	p38 FEP+ OPLS3e, ($N = 56$)	0.0104182	0.40631	Group 1 > Group 2
ptp1b QligFEPv2-Sage 2.2.1, ($N = 33$)	ptp1b FEP+ OPLS3e, ($N = 49$)	0.00116435	0.0523956	Group 1 > Group 2
thrombin QligFEPv2-Sage 2.2.1, ($N = 14$)	thrombin FEP+ OPLS3e, ($N = 16$)	0.683697	1	Group 1 > Group 2
tyk2 QligFEPv2-Sage 2.2.1, ($N = 22$)	tyk2 FEP+ OPLS3e, ($N = 24$)	0.36661	1	Group 1 > Group 2
bace QligFEPv2-Sage 2.2.1, ($N = 49$)	bace FEP+ OPLS3e, ($N = 58$)	0.506236	1	Group 1 > Group 2
cdk8 QligFEPv2-Sage 2.2.1, ($N = 55$)	cdk8 FEP+ OPLS3e, ($N = 54$)	0.0622233	1	Group 1 > Group 2
cmet QligFEPv2-Sage 2.2.1, ($N = 34$)	cmet FEP+ OPLS3e, ($N = 57$)	0.193378	1	Group 1 > Group 2
eg5 QligFEPv2-Sage 2.2.1, ($N = 38$)	eg5 FEP+ OPLS3e, ($N = 65$)	0.00149025	0.0655708	Group 1 > Group 2
hif2a QligFEPv2-Sage 2.2.1, ($N = 57$)	hif2a FEP+ OPLS3e, ($N = 92$)	0.0169982	0.645931	Group 1 > Group 2
shp2 QligFEPv2-Sage 2.2.1, ($N = 37$)	shp2 FEP+ OPLS3e, ($N = 56$)	0.000256992	0.0123356	Group 1 > Group 2
syk QligFEPv2-Sage 2.2.1, ($N = 59$)	syk FEP+ OPLS3e, ($N = 101$)	0.417356	1	Group 1 > Group 2
pfkfb3 QligFEPv2-Sage 2.2.1, ($N = 48$)	pfkfb3 FEP+ OPLS3e, ($N = 66$)	0.172559	1	Group 1 > Group 2
tnks2 QligFEPv2-Sage 2.2.1, ($N = 33$)	tnks2 FEP+ OPLS3e, ($N = 60$)	0.0640477	1	Group 1 > Group 2
cdk2 QligFEPv2-Sage 2.2.1, ($N = 22$)	cdk2 pmx-Sage 2.0, ($N = 25$)	0.546673	1	Group 1 < Group 2
cdk2 pmx-Sage 2.0, ($N = 25$)	cdk2 FEP+ OPLS3e, ($N = 25$)	0.977168	1	Group 1 < Group 2
jnk1 QligFEPv2-Sage 2.2.1, ($N = 27$)	jnk1 pmx-Sage 2.0, ($N = 31$)	0.348375	1	Group 1 < Group 2
jnk1 pmx-Sage 2.0, ($N = 31$)	jnk1 FEP+ OPLS3e, ($N = 31$)	0.698784	1	Group 1 < Group 2
mcl1 QligFEPv2-Sage 2.2.1, ($N = 60$)	mcl1 pmx-Sage 2.0, ($N = 71$)	0.932468	1	Group 1 < Group 2
mcl1 pmx-Sage 2.0, ($N = 71$)	mcl1 FEP+ OPLS3e, ($N = 71$)	0.702085	1	Group 1 < Group 2
p38 QligFEPv2-Sage 2.2.1, ($N = 51$)	p38 pmx-Sage 2.0, ($N = 56$)	0.996508	1	Group 1 < Group 2
p38 pmx-Sage 2.0, ($N = 56$)	p38 FEP+ OPLS3e, ($N = 56$)	0.34081	1	Group 1 < Group 2
ptp1b QligFEPv2-Sage 2.2.1, ($N = 33$)	ptp1b pmx-Sage 2.0, ($N = 49$)	0.742944	1	Group 1 < Group 2
ptp1b pmx-Sage 2.0, ($N = 49$)	ptp1b FEP+ OPLS3e, ($N = 49$)	0.999197	1	Group 1 < Group 2
thrombin QligFEPv2-Sage 2.2.1, ($N = 14$)	thrombin pmx-Sage 2.0, ($N = 16$)	0.174807	1	Group 1 < Group 2
thrombin pmx-Sage 2.0, ($N = 16$)	thrombin FEP+ OPLS3e, ($N = 16$)	0.801781	1	Group 1 < Group 2
tyk2 QligFEPv2-Sage 2.2.1, ($N = 22$)	tyk2 pmx-Sage 2.0, ($N = 24$)	0.153264	1	Group 1 < Group 2
tyk2 pmx-Sage 2.0, ($N = 24$)	tyk2 FEP+ OPLS3e, ($N = 24$)	0.865019	1	Group 1 < Group 2
cdk8 QligFEPv2-Sage 2.2.1, ($N = 55$)	cdk8 pmx-Sage 2.0, ($N = 54$)	0.8924	1	Group 1 < Group 2
bace QligFEPv2-Sage 2.2.1, ($N = 49$)	bace pmx-Sage 2.0, ($N = 58$)	0.925242	1	Group 1 < Group 2
bace pmx-Sage 2.0, ($N = 58$)	bace FEP+ OPLS3e, ($N = 58$)	0.0296713	1	Group 1 < Group 2
cdk8 pmx-Sage 2.0, ($N = 54$)	cdk8 FEP+ OPLS3e, ($N = 54$)	0.614805	1	Group 1 < Group 2
cmet QligFEPv2-Sage 2.2.1, ($N = 34$)	cmet pmx-Sage 2.0, ($N = 57$)	0.666658	1	Group 1 < Group 2
cmet pmx-Sage 2.0, ($N = 57$)	cmet FEP+ OPLS3e, ($N = 57$)	0.724097	1	Group 1 < Group 2
eg5 QligFEPv2-Sage 2.2.1, ($N = 38$)	eg5 pmx-Sage 2.0, ($N = 65$)	0.997929	1	Group 1 < Group 2
eg5 pmx-Sage 2.0, ($N = 65$)	eg5 FEP+ OPLS3e, ($N = 65$)	0.717374	1	Group 1 < Group 2
hif2a QligFEPv2-Sage 2.2.1, ($N = 57$)	hif2a pmx-Sage 2.0, ($N = 92$)	0.361865	1	Group 1 < Group 2
hif2a pmx-Sage 2.0, ($N = 92$)	hif2a FEP+ OPLS3e, ($N = 92$)	0.996469	1	Group 1 < Group 2
shp2 QligFEPv2-Sage 2.2.1, ($N = 37$)	shp2 pmx-Sage 2.0, ($N = 56$)	0.718005	1	Group 1 < Group 2

Group 1	Group 2	Original p-value	Corrected p-value	Test Direction
shp2 pmx-Sage 2.0, ($N = 56$)	shp2 FEP+ OPLS3e, ($N = 56$)	0.999446	1	Group 1 < Group 2
syk QligFEPv2-Sage 2.2.1, ($N = 59$)	syk pmx-Sage 2.0, ($N = 101$)	0.796044	1	Group 1 < Group 2
syk pmx-Sage 2.0, ($N = 101$)	syk FEP+ OPLS3e, ($N = 101$)	0.267672	1	Group 1 < Group 2
pfkfb3 QligFEPv2-Sage 2.2.1, ($N = 48$)	pfkfb3 pmx-Sage 2.0, ($N = 66$)	0.635787	1	Group 1 < Group 2
pfkfb3 pmx-Sage 2.0, ($N = 66$)	pfkfb3 FEP+ OPLS3e, ($N = 66$)	0.785128	1	Group 1 < Group 2
tnks2 QligFEPv2-Sage 2.2.1, ($N = 33$)	tnks2 pmx-Sage 2.0, ($N = 60$)	0.996206	1	Group 1 < Group 2
tnks2 pmx-Sage 2.0, ($N = 60$)	tnks2 FEP+ OPLS3e, ($N = 60$)	0.109195	1	Group 1 < Group 2
cdk2 QligFEPv2-Sage 2.2.1, ($N = 22$)	cdk2 FEP+ OPLS3e, ($N = 25$)	0.965838	1	Group 1 < Group 2
jnk1 QligFEPv2-Sage 2.2.1, ($N = 27$)	jnk1 FEP+ OPLS3e, ($N = 31$)	0.531063	1	Group 1 < Group 2
mcl1 QligFEPv2-Sage 2.2.1, ($N = 60$)	mcl1 FEP+ OPLS3e, ($N = 71$)	0.976634	1	Group 1 < Group 2
p38 QligFEPv2-Sage 2.2.1, ($N = 51$)	p38 FEP+ OPLS3e, ($N = 56$)	0.989753	1	Group 1 < Group 2
ptp1b QligFEPv2-Sage 2.2.1, ($N = 33$)	ptp1b FEP+ OPLS3e, ($N = 49$)	0.998872	1	Group 1 < Group 2
thrombin QligFEPv2-Sage 2.2.1, ($N = 14$)	thrombin FEP+ OPLS3e, ($N = 16$)	0.33124	1	Group 1 < Group 2
tyk2 QligFEPv2-Sage 2.2.1, ($N = 22$)	tyk2 FEP+ OPLS3e, ($N = 24$)	0.641636	1	Group 1 < Group 2
bace QligFEPv2-Sage 2.2.1, ($N = 49$)	bace FEP+ OPLS3e, ($N = 58$)	0.496258	1	Group 1 < Group 2
cdk8 QligFEPv2-Sage 2.2.1, ($N = 55$)	cdk8 FEP+ OPLS3e, ($N = 54$)	0.938516	1	Group 1 < Group 2
cmet QligFEPv2-Sage 2.2.1, ($N = 34$)	cmet FEP+ OPLS3e, ($N = 57$)	0.808864	1	Group 1 < Group 2
eg5 QligFEPv2-Sage 2.2.1, ($N = 38$)	eg5 FEP+ OPLS3e, ($N = 65$)	0.998543	1	Group 1 < Group 2
hif2a QligFEPv2-Sage 2.2.1, ($N = 57$)	hif2a FEP+ OPLS3e, ($N = 92$)	0.983185	1	Group 1 < Group 2
shp2 QligFEPv2-Sage 2.2.1, ($N = 37$)	shp2 FEP+ OPLS3e, ($N = 56$)	0.99975	1	Group 1 < Group 2
syk QligFEPv2-Sage 2.2.1, ($N = 59$)	syk FEP+ OPLS3e, ($N = 101$)	0.584024	1	Group 1 < Group 2
pfkfb3 QligFEPv2-Sage 2.2.1, ($N = 48$)	pfkfb3 FEP+ OPLS3e, ($N = 66$)	0.828903	1	Group 1 < Group 2
tnks2 QligFEPv2-Sage 2.2.1, ($N = 33$)	tnks2 FEP+ OPLS3e, ($N = 60$)	0.936953	1	Group 1 < Group 2

Table S2. Cycle closure-corrected binding affinity (ΔG_{bind}) estimation performance of QligFEP compared to pmx-Sage 2.0 (denoted as pmx) and FEP+ OPLS3e (denoted as OPLS3e) across 16 targets. Values show Kendall's τ, and mean unsigned error (MUE) with 95% confidence intervals represented as superscript and subscript numbers, respectively, calculated using 1000 bootstrapped samples. Ligand count represents the number of ligands involved in the perturbation network that had their binding affinities estimated from the RBFE values through the State Function-based Correction (SFC) algorithm.

	Kendall Tau			MUE			Ligand Count		
Target	OPLS3e	pmx	QligFEP	OPLS3e	pmx	QligFEP	OPLS3e	pmx	QligFEP
bace	$0.45_{0.22}^{0.64}$	$0.49_{0.3}^{0.65}$	$0.27_{0.06}^{0.48}$	$1.4_{1.1}^{1.73}$	$1.21_{0.99}^{1.43}$	$1.42_{1.11}^{1.75}$	36	36	36
cdk2	$0.44_{0.11}^{0.72}$	$0.56_{0.25}^{0.81}$	$0.57_{0.32}^{0.8}$	$0.69_{0.41}^{1.0}$	$0.66_{0.45}^{0.9}$	$1.16_{0.66}^{1.72}$	16	16	16
cdk8	$0.57_{0.38}^{0.75}$	$0.6_{0.46}^{0.72}$	$0.57_{0.37}^{0.75}$	$1.19_{0.9}^{1.5}$	$0.95_{0.76}^{1.14}$	$2.08_{1.63}^{2.56}$	33	33	32
cmet	$0.73_{0.56}^{0.85}$	$0.69_{0.4}^{0.89}$	$0.67_{0.48}^{0.82}$	$1.28_{0.88}^{1.63}$	$1.4_{1.01}^{1.91}$	$3.08_{1.95}^{4.27}$	24	24	24
eg5	$0.54_{0.37}^{0.71}$	$0.47_{0.24}^{0.67}$	$0.33_{0.09}^{0.55}$	$2.5_{2.18}^{2.81}$	$0.59_{0.42}^{0.77}$	$1.37_{0.98}^{1.79}$	28	28	27
hif2a	$0.45_{0.2}^{0.63}$	$0.36_{0.16}^{0.53}$	$0.38_{0.22}^{0.55}$	$0.85_{0.65}^{1.1}$	$1.23_{0.92}^{1.57}$	$1.97_{1.56}^{2.44}$	42	42	41
jnk1	$0.72_{0.53}^{0.87}$	$0.68_{0.51}^{0.84}$	$0.56_{0.29}^{0.77}$	$0.53_{0.38}^{0.69}$	$1.05_{0.7}^{1.38}$	$1.14_{0.81}^{1.51}$	21	21	21
mcl1	$0.42_{0.24}^{0.6}$	$0.47_{0.28}^{0.62}$	$0.47_{0.29}^{0.62}$	$1.1_{0.86}^{1.34}$	$1.15_{0.9}^{1.4}$	$1.57_{1.21}^{1.99}$	42	42	42
p38	$0.64_{0.46}^{0.8}$	$0.48_{0.28}^{0.66}$	$0.51_{0.32}^{0.66}$	$0.63_{0.46}^{0.81}$	$0.73_{0.52}^{0.97}$	$1.14_{0.79}^{1.54}$	34	34	34
pfkfb3	$0.6_{0.47}^{0.71}$	$0.47_{0.31}^{0.61}$	$0.55_{0.38}^{0.69}$	$1.08_{0.85}^{1.31}$	$1.02_{0.74}^{1.28}$	$1.5_{1.13}^{1.9}$	40	40	38
ptp1b	$0.77_{0.59}^{0.9}$	$0.3_{-0.07}^{0.61}$	$0.42_{0.09}^{0.65}$	$0.51_{0.34}^{0.71}$	$0.86_{0.55}^{1.22}$	$2.07_{1.38}^{2.72}$	23	23	23
shp2	$0.55_{0.34}^{0.73}$	$0.12_{-0.23}^{0.46}$	$0.54_{0.37}^{0.7}$	$0.79_{0.55}^{1.05}$	$1.58_{1.1}^{2.05}$	$2.49_{1.57}^{3.54}$	26	26	26
syk	$0.29_{0.07}^{0.5}$	$0.39_{0.18}^{0.58}$	$0.41_{0.19}^{0.6}$	$1.17_{0.94}^{1.4}$	$1.3_{0.97}^{1.64}$	$0.94_{0.71}^{1.21}$	44	44	44
thrombin	$0.56_{0.1}^{0.88}$	$0.45_{0.02}^{0.84}$	$0.45_{0.08}^{0.76}$	$0.58_{0.33}^{0.88}$	$1.04_{0.63}^{1.47}$	$0.89_{0.44}^{1.46}$	11	11	11
tnks2	$0.29_{-0.01}^{0.54}$	$0.34_{0.04}^{0.57}$	$0.02_{-0.25}^{0.3}$	$1.27_{0.83}^{1.73}$	$0.83_{0.6}^{1.08}$	$1.55_{1.1}^{2.02}$	27	27	27
tyk2	$0.73_{0.45}^{0.95}$	$0.63_{0.34}^{0.85}$	$0.63_{0.33}^{0.86}$	$0.62_{0.48}^{0.76}$	$0.83_{0.57}^{1.1}$	$0.53_{0.36}^{0.69}$	16	16	16
